# FKBP5 Orchestrates a Biphasic Microglial Response in Spinal Cord Injury by Sequentially Activating the GPR84/IL-1β Pathway and the LDHA–Lactylation–FXYD5/LGALS1 Axis

**DOI:** 10.34133/research.1384

**Published:** 2026-08-04

**Authors:** Haotian Li, Jing Wang, Daohui Li, Hangchuan Bi, Jin Yang, Junjie Dong, Zhiqiang Gong, Hongda Gong, Bing Wang, Lingqiang Chen

**Affiliations:** ^1^ Department of Orthopaedics, The First Affiliated Hospital of Kunming Medical University, Kunming, Yunnan 650032, China.; ^2^ Department of Rheumatology, The First People’s Hospital of Yunnan Province, The Affiliated Hospital of Kunming University of Science and Technology, Kunming, Yunnan 650032, China.

## Abstract

Spinal cord injury often causes permanent disability because the body’s own repair mechanisms are limited, and the molecules that control damage and healing are not fully understood. One such molecule, FK506-binding protein 5 (FKBP5), is known to rise sharply after injury, but whether it only drives harmful inflammation or also participates in later recovery has been unclear. In this study, we investigated how FKBP5 affects microglia—the brain’s immune cells—at different stages after spinal cord injury in mice. We found that FKBP5 plays a dual role. In the first few days, it works together with another protein, GPR84, to boost the production of an inflammatory signal called interleukin-1β. This signal pushes microglia into a destructive state and triggers a coordinated form of neuronal cell death that involves multiple death pathways. However, as FKBP5 levels continue to rise over time, it switches its function. It binds to and modifies an enzyme called LDHA, changing how microglia process lactate. This lactate then acts as a signal to add chemical tags (lactylation) onto histones, which turns on a protective gene, Fxyd5, and its partner Lgals1. These changes convert microglia from a harmful to a healing state, reduce neuronal death, and improve the local environment for tissue repair. Our results reveal that FKBP5 is a double-edged sword—first worsening damage, then promoting repair. This discovery suggests that precisely timing therapies that target FKBP5 could offer a new way to improve recovery after spinal cord injury.

## Introduction

Spinal cord injury (SCI) is a devastating neurological condition characterized by an initial mechanical insult, followed by a cascade of secondary pathological events, which often lead to permanent motor, sensory, and autonomic dysfunctions [1]. The intricate interplay between neuroinflammation, neuronal death, and tissue repair plays a pivotal role in determining the functional outcomes after SCI [2]. Central to this process are the microglia, which are the resident immune cells of the central nervous system, as they exhibit remarkable plasticity and can adopt either detrimental pro-inflammatory or beneficial anti-inflammatory phenotypes in response to dynamic microenvironmental cues [3].

Recent advances have highlighted the multifaceted role of FK506-binding protein 5 (FKBP5), a co-chaperone of the heat shock protein 90 complex, in cellular stress responses and inflammatory regulation [4]. In the acute phase of SCI, the expression of FKBP5 is rapidly up-regulated, activating the NF-κB signaling axis and driving microglial polarization toward a pro-inflammatory phenotype [5]. This inflammatory shift exacerbates secondary injury by promoting coordinated activation of pyroptosis, apoptosis, and necroptosis pathways—a process we cautiously describe as “PANoptosis-like” cell death, as definitive demonstration of the complete PANoptosome complex (e.g., ZBP1 and cFLIP) requires further investigation [6]. However, the role of FKBP5 in the subacute and chronic phases of SCI, particularly its potential involvement in tissue repair and immune modulation, remains poorly understood.

Emerging evidence suggests that metabolic reprogramming, particularly in lactate metabolism, plays a crucial role in shaping microglial function and neuronal survival [7]. Lactate, once considered a mere metabolic waste product, is now recognized as a key signaling molecule influencing epigenetic modifications, including histone lactylation [8]. These modifications can regulate the gene expression patterns, thereby modulating cellular phenotypes and tissue responses. Nevertheless, the interplay between FKBP5, lactate metabolism, and epigenetic regulation in the context of SCI has not been systematically investigated.

In the present study, we identified a biphasic regulatory mechanism of FKBP5 in SCI repair, with clearly delineated acute-phase and subacute-phase functions. During the acute phase, FKBP5 promotes microglial pro-inflammatory polarization and neuronal PANoptosis-like neuronal death through the GPR84/interleukin-1 beta axis. As the injury progresses, the sustained elevation of FKBP5 reprograms lactate metabolism by modulating LDHA phosphorylation at Tyr10, leading to increased histone H4K12 lactylation at the Fxyd5 promoter region. This epigenetic activation of the FXYD5/LGALS1 signaling pathway drives microglial transition into an anti-inflammatory phenotype, suppresses PANoptosis-like cell death, and optimizes the local immune microenvironment, ultimately facilitating tissue repair. Through single-cell ligand–receptor communication analysis, we also identified enriched interactions such as GPR37L1–PSAP and GPR37–PSAP as exploratory observations; however, unlike the mechanistically validated FKBP5–GPR84/IL-1β and FKBP5–LDHA–lactylation–FXYD5/LGALS1 axes, the functional integration of GPR37/PSAP signaling with the FKBP5–lactylation pathway requires future validation and is therefore presented as an auxiliary finding. Our study findings provide novel insights into the dual roles of FKBP5 in pathogenesis and repair of SCI, highlighting its potential as a therapeutic target for the treatment of this condition. The discovery of this FKBP5-mediated metabolic–epigenetic regulatory axis not only advances our understanding of the pathophysiology of SCI but also opens new avenues for developing targeted therapies that harness the reparative potential of microglial phenotypic switching. We acknowledge that our conclusions regarding PANoptosis are based on pharmacological inhibition and co-immunoprecipitation (co-IP) evidence of multipathway activation rather than definitive demonstration of the complete PANoptosome; accordingly, we describe this phenomenon as “PANoptosis-like” throughout the manuscript.

## Results

### Important changes during the repair process following SCI

**Fig. 1.**
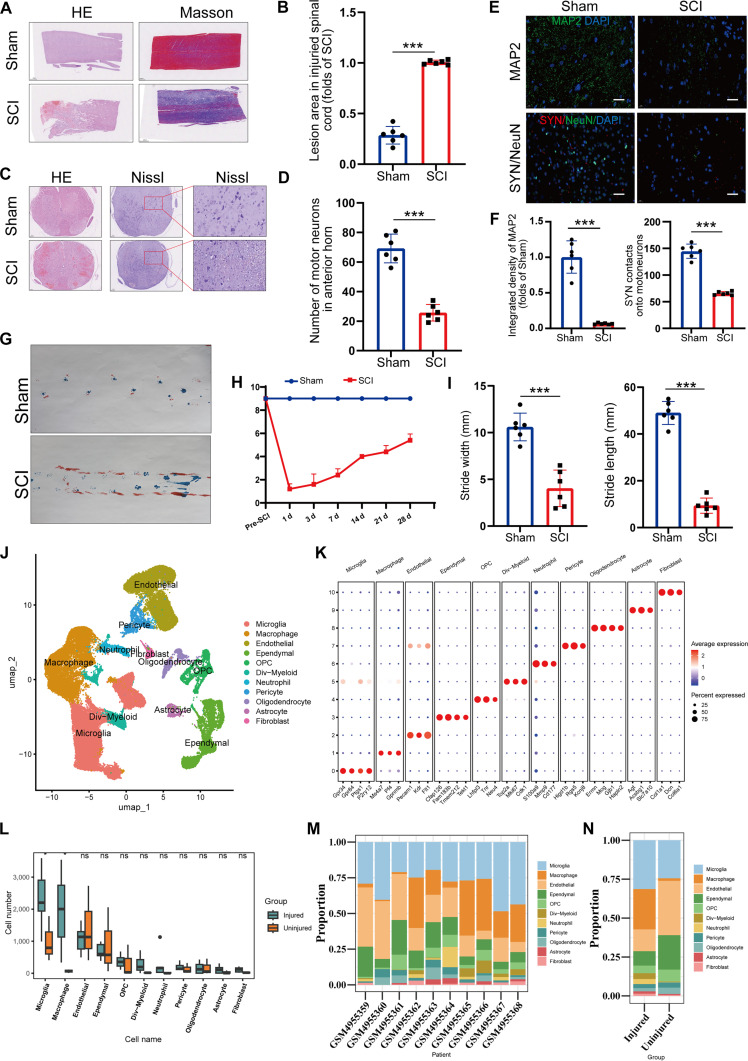
Single-cell immune RNA sequencing reveals an increased proportion of microglial cells in spinal cord injury. (A) Longitudinal spinal cord sections from the indicated groups were subjected to Masson and HE staining at 28 dpi (scale bar = 250 μm). (B) Quantitative analysis of Masson staining in the spinal cord tissues across different groups. (C) At 14 dpi, transverse spinal cord sections from the indicated groups were analyzed using HE and Nissl staining (scale bar = 100 μm). (D) Quantification of Nissl-positive motor neurons in the anterior horn of the spinal cord for the indicated groups. (E) Images showing spinal cord sections incubated with anti-MAP2 antibodies, and SCI sections stained with anti-SYN/NeuN antibodies collected on day 28 (scale bar = 20 μm). (F) Measurement of MAP2 optical density at 28 dpi, along with a quantitative analysis of neuron-contacting synapses. (G) Photographs of mouse footprints taken at 28 dpi. (H) BMS scores of the indicated groups at various time points. (I) Values representing stride length and width for each group of mice. (J) UMAP plot illustrating single-cell population annotations. (K) Cell-type annotations of cell clusters based on the marker genes. (L) Differential cells between spinal cord injury (shown in blue) and noninjured conditions (shown in yellow). (M) Box plots showing the proportions of cell types across different samples. (N) Box plots comparing the proportions of cell types between injured and noninjured spinal cords. *n* = 6; data are presented as mean ± standard error of the mean (SEM); ***P < 0.001.

A marked rise in microglial cell count was detected during the recovery phase after SCI. To evaluate the motor function changes post-SCI, the present study employed various methodologies, including hematoxylin and eosin (HE), Nissl, Masson’s trichrome, and immunofluorescence (IF) staining; footprint analysis; and Basso Mouse Scale (BMS) scoring system for locomotor performance. Histological assessments revealed that, compared to the sham group, the SCI animals exhibited more pronounced glial scar formation at the injury site (Fig. 1A and B). Moreover, a notable reduction in motor neuron count was observed in the SCI group (Fig. 1C and D). The IF staining results further confirmed a substantial reduction in the MAP2 expression levels and number of SYN-positive synapses on the neurons within the injured area (Fig. 1E and F). Functional assessment findings revealed that, at multiple time points following injury—namely, 1, 3, 7, 14, 21, and 28 days—the BMS scores in the sham group were markedly elevated compared to those in the SCI group (Fig. 1G to I).

To delve deeper into cellular heterogeneity during the repair process of SCI, we reanalyzed the Gene Expression Omnibus (GEO) dataset (GSE162610). Utilizing Uniform Manifold Approximation and Projection (UMAP) projection for visualization, 11 distinct cell subpopulations with unique temporal dynamics were identified (Fig. 1J). The UMAP distribution of each cell subpopulation along with their biological replicates is shown in Fig. S1A to D. A differential expression gene (DEG) analysis provided distinctive molecular signatures for each cell type (Fig. 1K and Fig. S1E and F), with some of which diverging from the classical markers reported in previous studies (Fig. S2). By comparing the cell clusters between the sham and SCI groups, marked alterations in the number of microglial cells before and after injury were noted (Fig. 1L). A further temporal analysis uncovered dynamic shifts in the microglial populations, revealing an early increase followed by a gradual decline toward baseline (Fig. 1M and N and Fig. S1G and H). These results provide an essential basis for further detailed investigations into how microglia contribute to the biphasic mechanisms involved in SCI repair—an initial pro-inflammatory phase and a subsequent reparative phase.

### Differential responses of the microglia/macrophages to SCI

Subsequently, we investigated the injury site during the early phases after SCI. By 1 to 2 days postinjury (dpi), a distinct separation was observed between the 2 ends of the damaged tissue, and serotonergic axons were found to end proximal to the lesion site (Fig. S3A). By 3 dpi, cells expressing fibronectin (fibronectin+) were observed within the lesion site (Fig. S3A); by 7 dpi, the gap had closed, the fibronectin signal decreased, and serotonergic axons grew across the injury site. To determine the origin of the fibronectin-positive cells present in the lesion gap at 3 dpi, Cx3cr1-GFP transgenic mice—featuring GFP-labeled microglia—were employed. By 2dpi, the microglia accumulated at the stump ends, losing P2Y12 expression and instead expressing markers indicative of activated microglia, including SPP1+ and CD68 (Fig. S3B). Morphologically, these cells transitioned from highly ramified to amoeboid shapes (Fig. S3B), in line with the activation observed during injury response.

To investigate the role of microglia, PLX5622, a colony-stimulating factor 1 receptor (CSF1R) inhibitor, was used to deplete the microglia. Strikingly, the depletion of the microglia hindered bridge formation between the 2 stumps at 3 or 7 dpi (Fig. S3C and D). Starting at 3 dpi, activated microglia were observed in the lesion site where fibronectin-positive bridges connected the stumps, in the absence of glial fibrillary acidic protein (GFAP)-positive astrocytes and collagen I-expressing fibroblasts (Fig. S4A). Interestingly, these cells began to re-express P2Y12 while maintaining positivity for SPP1 and CD68 (Fig. S3E). By approximately 7 dpi, the majority of microglial cells exhibited a ramified morphology and expressed P2Y12, while showing no immunoreactivity for SPP1 or CD68 (Fig. S3E).

In conjunction with the observations of minimal cell death (Fig. S4B) and the short time frame for cell proliferation, our findings indicate that microglia, initially activated after SCI, rapidly return to a homeostatic state within the first postinjury week. This highlights the critical role and dynamic nature of microglia in the repair of SCI.

### SCI (1 dpi) induces pro-inflammatory phenotypes in the microglia, leading to neuronal PANoptosis-like cell death

**Fig. 2.**
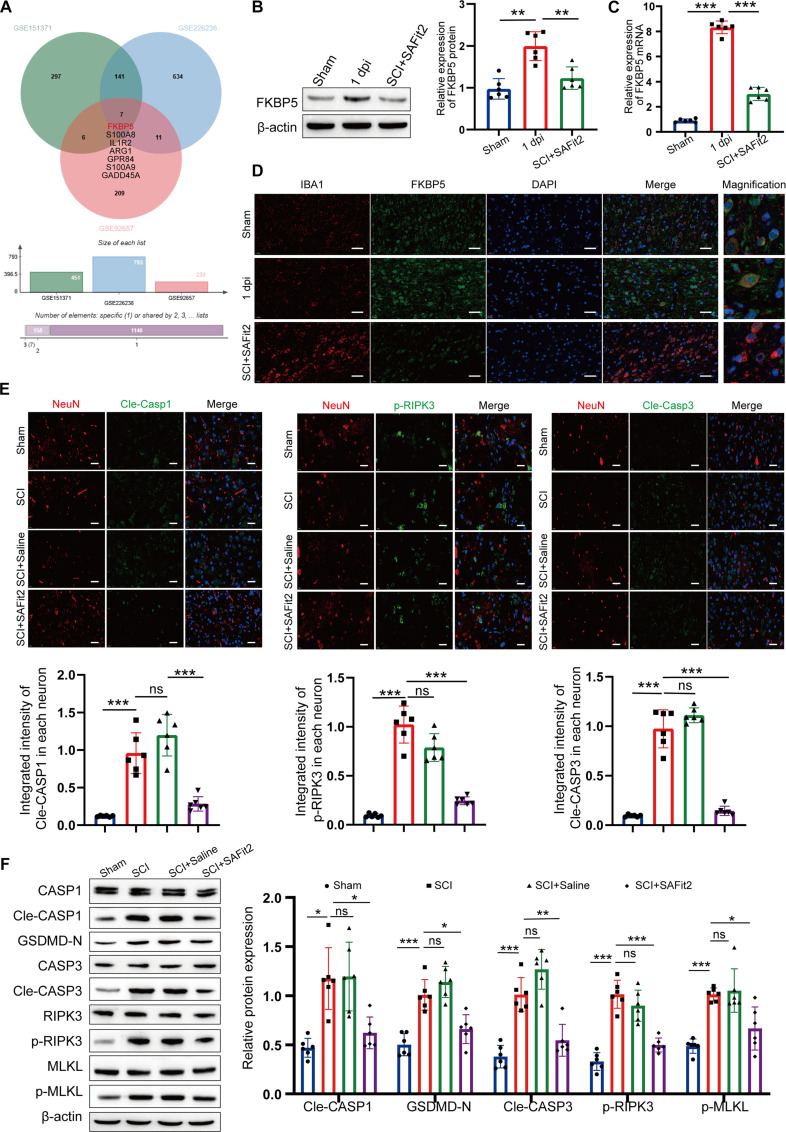
Microglia regulate PANoptosis-like cell death post-spinal cord injury via FKBP5. (A) Venn diagram analysis using GEO datasets (GSE151371, GSE226238, and GSE92657) to identify the overlapping genes. (B) Western blot and quantification of FKBP5 expression in the spinal cord tissues at 1 dpi. (C) qPCR analysis of FKBP5 mRNA levels in the spinal cord tissues at 1 dpi. (D) Immunofluorescence images showing cellular localization of FKBP5 in the spinal cord tissue at 1 dpi (scale bar = 20 μm). (E) Immunohistochemical staining and quantitative analysis of Cle-CASP1, p-RIPK3, and Cle-CASP3 in the neurons from the thoracic spinal cord sections at 1 dpi (scale bar = 20 μm). (F) Western blot and quantification of PANoptosis-related protein expression in the spinal cord tissue at 1 dpi. *n* = 6; data are presented as mean ± SEM; *P < 0.05; **P < 0.01; ***P < 0.001. ns, not significant.

To further elucidate the mechanisms of neuronal cell death following SCI, we identified the FKBP5 protein through an analysis of GEO datasets (GSE151371, GSE226238, and GSE92657) (Fig. 2A). To assess whether in vivo treatment with an FKBP5 inhibitor (10 mg/kg SAFit2) could influence the levels of FKBP5 mRNA and protein expressions, we performed Western blot and quantitative polymerase chain reaction (qPCR) analyses. These revealed that the FKBP5 mRNA and protein expressions were markedly increased at 1 dpi, but treatments with inhibitors effectively reduced these levels (Fig. 2B and C). Additionally, colocalization studies using FKBP5 and IBA1 confirmed the presence of FKBP5 within the microglia (Fig. 2D).

Earlier studies have proposed PANoptosis, a newly identified type of programmed cell death that involves intricate crosstalk among pyroptosis, apoptosis, and necroptosis [9]. Notably, prior investigations have indicated that SCI activates the pathways associated with pyroptosis, apoptosis, and necroptosis [10,11]. Thus, we hypothesized that coordinated activation of these 3 pathways—a PANoptosis-like process—may occur following SCI, potentially mediated by FKBP5. To investigate this possibility, we performed the following experiments. In primary cultured neurons, we observed distinct ASC protein aggregates (specks) via immunofluorescence staining under SCI-mimicking conditions (Fig. S5A and B). Furthermore, our co-IP experiments confirmed that in spinal cord tissue lysates at 1 dpi, there is a physical interaction between RIPK3 and both Caspase-8 and NLRP3 (Fig. S5C). This result provides supporting evidence for the formation of a multiprotein complex containing core components of both necroptosis and apoptosis, a hallmark feature of the PANoptosome. To further assess pathway-level involvement, we used a pyroptosis inhibitor (VX-765, a Caspase-1 inhibitor), an apoptosis inhibitor (Z-VAD-FMK, a pan-Caspase inhibitor), and a necroptosis inhibitor (Nec-1s, a RIPK1 inhibitor). The results showed that treatment with any single inhibitor only partially alleviated SCI-induced neuronal death. Treatment with dual inhibitors showed additive but incomplete rescue, whereas simultaneous inhibition of all 3 pathways (VX-765 + Z-VAD-FMK + Nec-1s) provided the maximum rescue of neurons (Fig. S5D and E). Of note, while these pharmacological data strongly support the coordinated involvement of all 3 cell death pathways, we acknowledge that Z-VAD-FMK is a pan-caspase inhibitor rather than a specific apoptosis effector blocker, and Nec-1s targets RIPK1 rather than the terminal necroptosis effector MLKL. Additionally, core PANoptosome components such as ZBP1 and cFLIP were not examined in this study. Our IF results demonstrated that reducing the FKBP5 expression mitigated Cle-CASP1, Cle-CASP3, and p-RIPK3 activations in the neurons, whereas increased FKBP5 expressions promoted their activations (Fig. 2E). Western blot analysis showed that inhibiting FKBP5 expression markedly decreased the Cle-CASP1, GSDMD-N, Cle-CASP3, p-RIPK3, and p-MLKL levels (Fig. 2F). Furthermore, fluorescence staining revealed that the SCI-induced treatments triggered apoptosis (terminal deoxynucleotidyl transferase-mediated deoxyuridine triphosphate nick end labeling [TUNEL] staining; Fig. S6A) and necroptosis (propidium iodide [PI] staining; Fig. S6B). Pretreatment with the FKBP5 inhibitor substantially reversed the regulated cell death morphology induced by SCI (Fig. S6A and B).

**Fig. 3.**
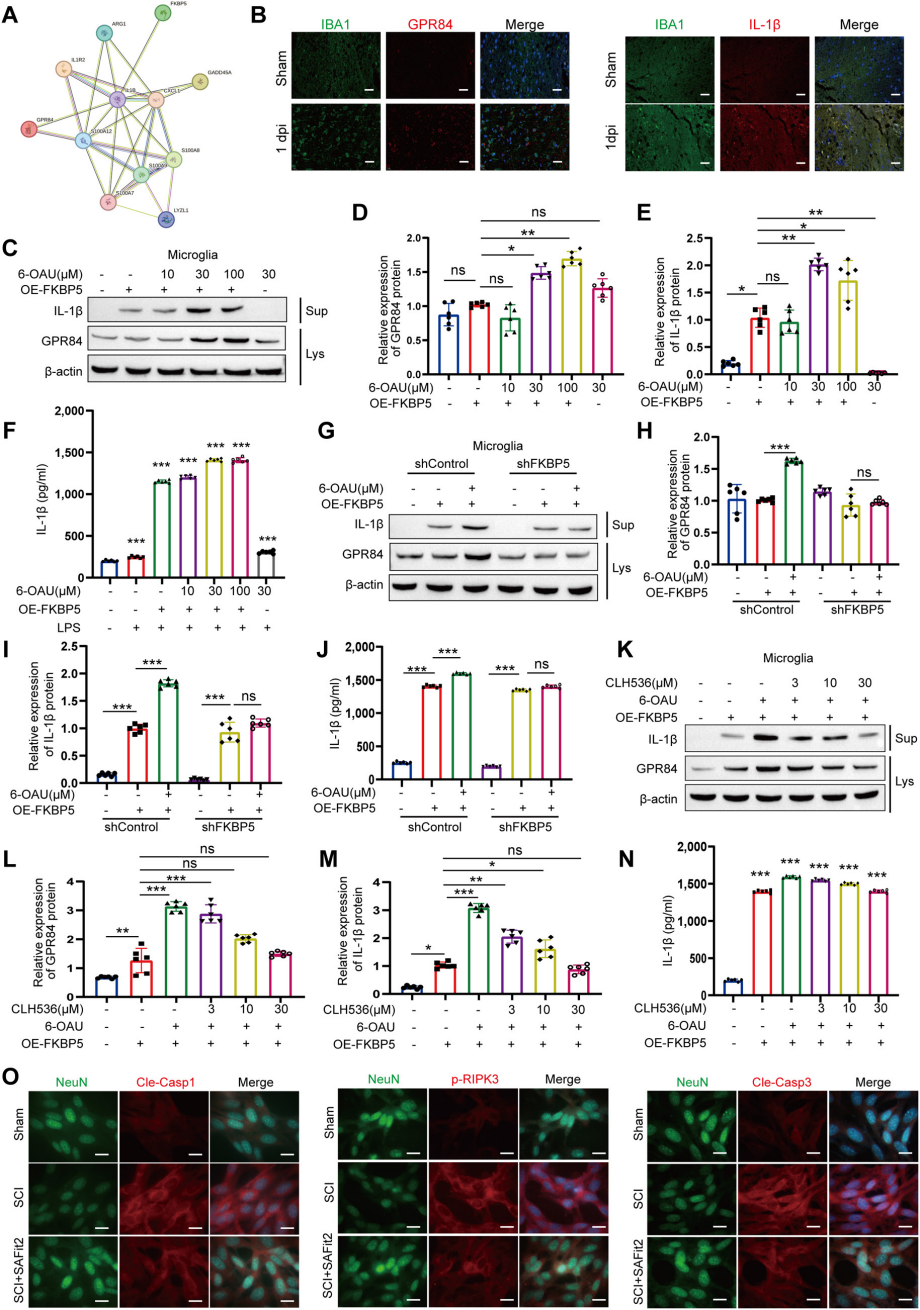
Regulation of PANoptosis-like cell death post-spinal cord injury by FKBP5/GPR84/IL-1β. (A) Interaction analysis among FKBP5, GPR84, and IL-1β using the STRING database. (B) Immunofluorescence images showing the colocalization of GPR84 and IL-1β with IBA1, indicating their localization in the microglia (scale bar = 20 μm). (C) Representative Western blot analysis showing IL-1β expressions in the culture supernatants (Sup) and GPR84 in the lysate (Lys) of microglial cells pretreated with LPS for 3 h and then stimulated with various concentrations of 6-OAU and FKBP5 overexpression. (D and E) Quantification of GPR84 (D) and IL-1β (E) protein levels from Western blotting in (C). The proteins were normalized to GAPDH in the same sample. (F) ELISA analysis of IL-1β levels in the supernatants of microglial cells pretreated with LPS for 3 h and then stimulated with different concentrations of 6-OAU and FKBP5 overexpression. (G) Representative Western blot analysis showing the expression of IL-1β in the culture supernatants (Sup) and GPR84 in the lysate (Lys) of microglial cells pretreated with LPS for 3 h, either in the control (NC) or shFKBP5 knockdown group, and then stimulated with FKBP5 overexpression and 6-OAU (30 μM). (H and I) Quantification of GPR84 (H) and IL-1β (I) protein levels from Western blotting in (G). Proteins were normalized to GAPDH in the same sample. (J) ELISA analysis of IL-1β levels in the supernatants of microglial cells pretreated with LPS for 3 h, either in the control (NC) or shFKBP5 knockdown group, and then stimulated with FKBP5 overexpression and 6-OAU (30 μM). (K) Representative Western blot analysis showing IL-1β expressions in the culture supernatants (Sup) and GPR84 in the lysate (Lys) of microglial cells pretreated with LPS and then stimulated with FKBP5 overexpression and 6-OAU (30 μM), in the presence of various CLH536 doses. (L and M) Quantification of GPR84 (L) and IL-1β (M) protein levels from Western blotting in (K). Proteins were normalized to GAPDH in the same sample. (N) ELISA test of IL-1β levels in the supernatants of microglial cells pretreated with LPS and then stimulated with FKBP5 overexpression and 6-OAU (30 μM), in the presence of various CLH536 doses. (O) Primary neurons treated with a normal microglial cell culture medium (Sham), pro-inflammatory microglial cell supernatant (SCI), or pro-inflammatory microglial cell supernatant treated with an FKBP5 inhibitor (SCI + SAFit2). Immunofluorescence was used to examine the PANoptosis-like phenotype (c-caspase-3, c-caspase-1, and p-RIPK3) (scale bar = 5 μm). *n* = 6; data are presented as mean ± SEM; *P < 0.05; **P < 0.01; ***P < 0.001. ns, not significant.

To explore the mechanisms by which FKBP5 regulates this coordinated cell death response, we conducted a protein–protein interaction (PPI) analysis using proteins identified through the intersection of GEO dataset results, uncovering the interactions among FKBP5, GPR84, and interleukin-1 beta (IL-1β) (Fig. 3A). Co-IP experiments verified these interactions (Fig. S6C and D). Both GPR84 and IL-1β were localized within the microglia (Fig. 3B). To explore the regulatory relationships further, we overexpressed FKBP5 in the primary microglial cells pretreated with lipopolysaccharide (LPS) and stimulated them with the GPR84 agonist 6-OAU, evaluating inflammasome activation via IL-1β production. While 6-OAU by itself did not trigger IL-1β release, it markedly amplified IL-1β secretion in cells overexpressing FKBP5, with the enhancement increasing in a dose-dependent fashion (Fig. 3C to F). In the FKBP5 knockout microglial cells, overexpression of FKBP5 continued to promote IL-1β production, whereas the potentiating effect of 6-OAU was no longer observed (Fig. 3G to J). Furthermore, the enhancement of IL-1β production in microglial cells overexpressing FKBP5 induced by 6-OAU was suppressed by the GPR84 antagonist CLH536 in a concentration-dependent manner (Fig. 3K to N). These findings suggest that FKBP5 collaborates with GPR84 to activate IL-1β.

Subsequently, we cultured the primary neurons in conditioned media from the differently treated microglial cells and observed the outcomes consistent with our in vivo experiments (Fig. 3O), underscoring the role of IL-1β in inducing PANoptosis-like neuronal death.

In summary, SCI leads to elevated FKBP5 expressions in the microglia, which, together with GPR84, activates IL-1β. The subsequent secretion of IL-1β into the extracellular environment induces coordinated pyroptosis, apoptosis, and necroptosis pathway activation—a PANoptosis-like phenotype—in neurons. Although our data provide robust evidence for multipathway involvement, we cautiously describe this phenomenon as “PANoptosis-like” given that definitive demonstration of the complete PANoptosome complex (e.g., ZBP1 and cFLIP) was not performed. The present study highlights the critical involvement of FKBP5 in the inflammatory response following SCI and its potential as a therapeutic target for preventing this coordinated cell death process.

### Microglia elevate overall lactylation levels through FKBP5/LDHA

**Fig. 4.**
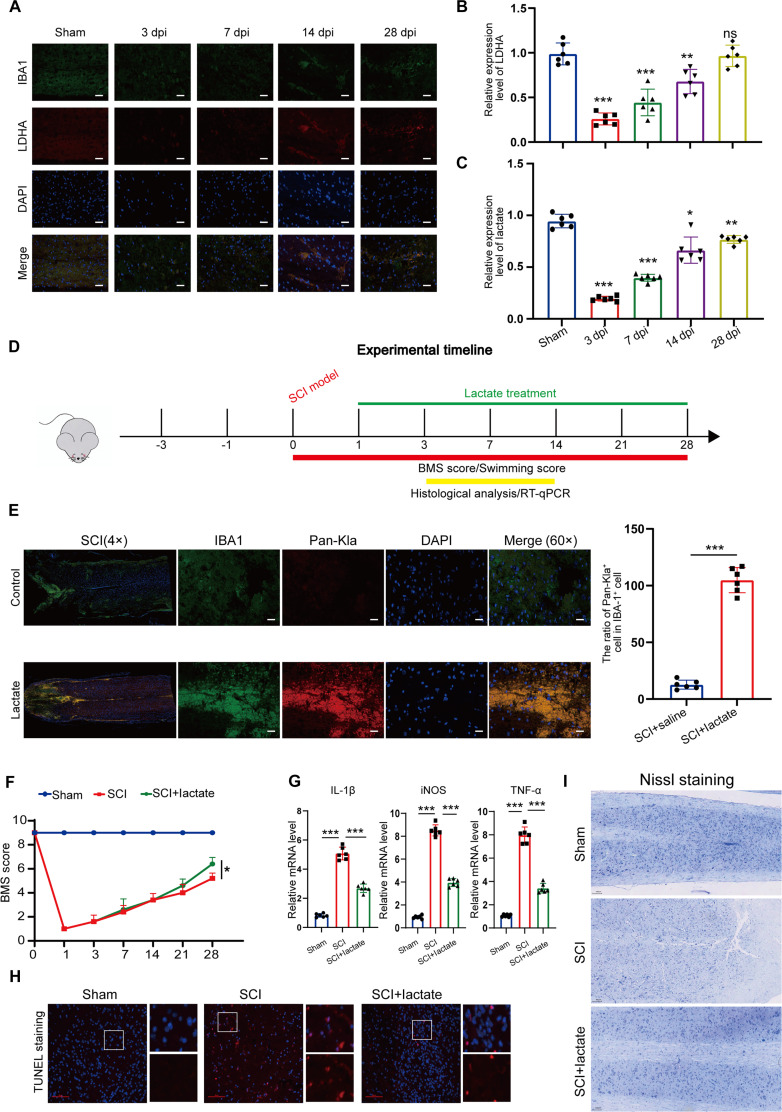
Microglial enhancement of SCI recovery through altered glycolysis and lactate metabolism. (A) Immunofluorescence results showing IBA1 and LDHA expressions in mice (scale bar = 20 μm). (B) Relative immunofluorescence intensity of LDHA within the microglial cells. (C) Lactate levels in the serum and spinal cord tissue of mice at various time points post-SCI. (D) Schematic representation of the experimental design involving mice. (E) Immunofluorescence findings in the spinal cord, including a semiquantitative analysis of Pan-Kla fluorescence intensity (scale bar = 20 μm). (F) BMS scores of mice at different time intervals posttreatment. (G) Changes in the pro-inflammatory phenotypes of the microglia following lactate treatment, as assessed by qPCR. (H) TUNEL staining and quantitative analysis of apoptosis-positive cells (scale bar = 100 μm). (I) Nissl staining and quantitative assessment of Nissl-positive neurons (scale bar = 250 μm). *n* = 6; data are presented as mean ± SEM; *P < 0.05; **P < 0.01; ***P < 0.001. ns, not significant.

To investigate microglia’s involvement in SCI recovery, earlier research has emphasized the neuroprotective benefits associated with lactate-induced lactylation of microglia-related proteins during SCI [12]. Moreover, the Kyoto Encyclopedia of Genes and Genomes enrichment analysis conducted on differentially expressed genes indicated marked changes in signaling pathways associated with glycolysis (Fig. S7A). We conducted IF analyses on spinal cord tissues before injury and at 3, 7, 14, and 28 dpi. Our findings indicated that the glycolysis-associated gene expression was markedly down-regulated at 3 and 7 dpi compared to the preinjury levels, with partial recovery observed by 14 and 28 dpi (Fig. 4A). The IF and semiquantitative analyses demonstrated that the LDHA intensity and lactate levels were notably restored in the 14 and 28 dpi groups, compared to the 3 and 7 dpi groups (Fig. 4B and C).

To explore the influence of lactate on functional restoration and the inflammatory microenvironment following SCI, we administered lactate treatment following SCI in mice to observe its impact on injury repair (Fig. 4D). Mice were allocated into 3 groups: sham, SCI, and SCI with lactate administration. Immunofluorescence staining revealed that the percentage of Pan-Kla-positive cells among IBA-1-positive cells was markedly increased in the SCI mice treated with lactate compared to those in the untreated SCI group (Fig. 4E). Motor performance was evaluated through BMS scoring and swimming assessments. No marked differences in these measures were observed at 1 dpi, indicating consistent SCI severity across groups (Fig. 4F). However, at 14, 21, and 28 dpi, the SCI + lactate group exhibited markedly higher BMS scores than the SCI group (Fig. 4F). Extraction of mRNA from spinal cord tissues collected at 3 dpi demonstrated that lactate markedly attenuated the up-regulation of IL-1β, inducible nitric oxide synthase (iNOS), and tumor necrosis factor-alpha (TNF-α) mRNA levels induced by SCI (Fig. 4G). TUNEL staining to detect apoptosis-positive cells revealed a markedly increased proportion in the SCI group compared to the sham group, whereas this elevation was notably reversed in the SCI + lactate group (Fig. 4H). Nissl staining revealed that SCI led to a reduction in neuronal count within the spinal cord, a deficit that was markedly restored in the SCI + lactate group (Fig. 4I). These results indicate a potential link between lactylation and neuroinflammatory processes following SCI.

To further confirm the possible involvement of lactylation in modulating neuroinflammatory processes, primary microglial cells were treated with interferon γ (IFNγ) and LPS to mimic secondary neuroinflammation after SCI in mice. Subsequent lactate treatment promoted lactylation, which was further enhanced by Lac or S-Lac (sodium lactate) (Fig. S7B and C). Consistent trends in the lactate levels were also observed (Fig. S7D). The evaluation of microglial polarization states showed increased pro-inflammatory marker (IL-1β, iNOS, and TNF-α) and decreased anti-inflammatory marker (Arg-1, CD206, and IL-10) levels in the IFNγ + LPS group, indicative of activated inflammation (Fig. S7E). Compared with the IFNγ + LPS group, both the IFNγ + LPS + Lac and IFNγ + LPS + S-Lac groups exhibited markedly reduced mRNA expression of pro-inflammatory markers, along with increased levels of anti-inflammatory markers (Fig. S7E), demonstrating that the Lac or S-Lac treatment suppressed the pro-inflammatory phenotypes and activated the anti-inflammatory phenotypes. The IF staining results for iNOS showed that the elevated iNOS intensity due to the IFNγ + LPS treatment was reduced by Lac or S-Lac treatment, whereas the lowered CD206 intensity was markedly increased (Fig. S7F and G). The levels of pro-inflammatory marker proteins increased in the IFNγ + LPS group, but were markedly decreased following treatment with Lac or S-Lac (Fig. S7H and I). Conversely, the levels of anti-inflammatory marker proteins were markedly increased following treatment with Lac or S-Lac (Fig. S7H and I). These results indicate that the Lac or S-Lac treatment can promote an anti-inflammatory phenotype in the microglia, thereby exerting an anti-inflammatory effect.

**Fig. 5.**
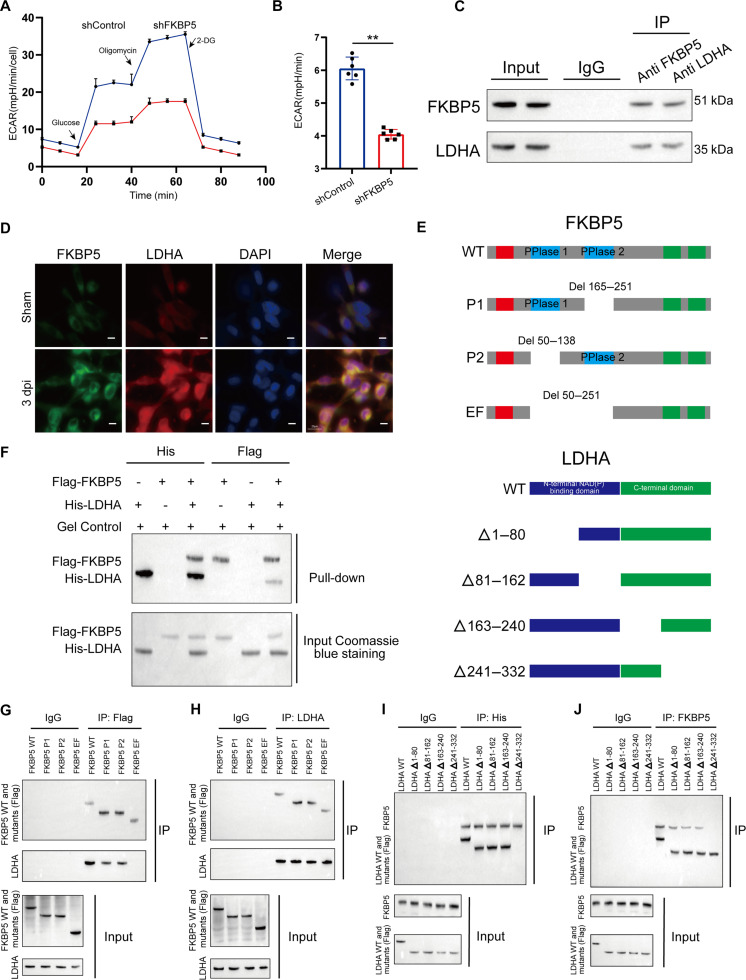
FKBP5 directly binds to LDHA to regulate lactate metabolism. (A and B) Extracellular acidification rates (ECARs) in the microglial cells following FKBP5 knockdown. (C) Co-immunoprecipitation (co-IP) analysis of the interaction between endogenous FKBP5 and LDHA in the microglial cells. (D) Cellular localization of FKBP5 and LDHA determined by immunofluorescence microscopy (scale bar = 5 μm). (E) In vitro pull-down assays demonstrating that FKBP5 directly binds to LDHA. (F) Schematic representation of FKBP5 deletion mutants, highlighting various domains such as the signal peptide (red), PPlase (blue), and EF-hand motif (green). Moreover, a schematic of LDHA deletion mutants with N-terminal regions marked in blue and C-terminal regions in green is also shown. (G and I) Co-IP experiments conducted in HEK293 cells to analyze the interaction between exogenous FKBP5 and various LDHA fragments or between exogenous LDHA and different FKBP5 mutants. IgG was used as a negative control for immunoprecipitation. (H and J) Co-IP analyses performed in the microglial cells to investigate the interactions between different regions of FKBP5 and endogenous LDHA or between various regions of LDHA and endogenous FKBP5. IgG served as the negative control. Data are presented as mean ± SEM; **P < 0.01.

A previous study has indicated that FKBP10 may play a crucial role in glycolysis regulation [13]. Given the similar domains within the FKBP protein family, we hypothesized that FKBP5 regulates the glycolytic processes in the microglia following SCI. We observed that, employing a Seahorse XF24 analyzer, knocking down the FKBP5 expression markedly inhibited the extracellular acidification rate in the microglial cells (Fig. 5A and B), reflecting a reduced glycolytic capacity. Conversely, the rate of oxygen consumption, reflecting mitochondrial oxidative phosphorylation activity, was markedly elevated (Fig. S8A and B). Further confirmation came from the functional assays demonstrating the interaction with LDHA. The co-IP experiments with endogenously expressed FKBP5 and LDHA in the microglial cells confirmed their interaction (Fig. 5C). Additionally, IF colocalization showed the coexpression of FKBP5 and LDHA in the microglial cells (Fig. 5D). In vitro His- and Flag-pulldown assays also demonstrated direct binding between FKBP5 and LDHA (Fig. 5E).

To identify the critical regions responsible for their interaction, FKBP5 was split into 3 segments, each tagged with a Flag epitope, while LDHA was separated into 4 parts, each fused with a His tag, in order to more precisely map the interaction region (Fig. 5F). The co-IP results revealed that exogenous LDHA could bind to all FKBP5 fragments, except FKBP5-EF (Del 50 to 251) in the 293T cells (Fig. 5G). Exogenous FKBP5 could bind to all the LDHA fragments, except LDHA-Δ241-332 (Fig. 5I). These results provide initial evidence that FKBP5 directly interacts with the C-terminal domain of LDHA (residues 241 to 332) and associates with LDHA via its 2 PPIase domains, indicating that even one PPIase domain alone can mediate this binding. Subsequent co-IP experiments in the microglial cells supported these conclusions (Fig. 5H and J).

Western blot results indicated that the protein levels of LDHA and LDHB remained unchanged following FKBP5 knockdown in microglial cells (Fig. S8C). The activity of lactate dehydrogenase and the levels of lactate generation were assessed in microglia, revealing marked enhancement or inhibition upon the overexpression or knockdown of FKBP5 (Fig. S8D and E). This indicates that FKBP5 regulates the LDHA enzyme activity, rather than its protein expression. The LDHA enzyme activity is influenced by phosphorylation at Tyr10, which enhances tetramer formation, or phosphorylation at Tyr38, increasing the LDHA’s binding affinity for NADH. Additionally, the Y10 phosphorylation of LDHA is catalyzed by FGFR1 [14]. Gel filtration chromatography demonstrated a marked reduction in LDHA tetramer abundance upon FKBP5 knockdown (Fig. S8F), indicating that FKBP5 promotes the active tetramer formation, consistent with its role in protein folding and spatial structure formation. Western blot analysis further demonstrated that overexpression or knockdown of FKBP5 increased or decreased phosphorylation at the LDHA-Y10 site, respectively, while having no effect on the protein levels of LDHA or LDHB (Fig. S8G). The treatment of microglial cells in the presence of the FGFR1 inhibitor AZD4547 (500 nM, 24 h) revealed that the overexpressed FKBP5 could not enhance LDHA-Y10 phosphorylation (Fig. S8H). In vitro kinase and Western blot analyses demonstrated that FKBP5 no longer regulates LDHA-Y10 phosphorylation when FGFR1 is absent (Fig. S8I). Collectively, these results suggest that FKBP5 promotes LDHA-Y10 phosphorylation in an FGFR1-dependent manner. Further validation using tissue microarrays confirmed the correlation between FKBP5 expression and LDHA-Y10 phosphorylation (Fig. S8J). These results suggest that FKBP5 may modulate LDHA protein activity by controlling its phosphorylation state.

### Microglial histone lactylation up-regulation inhibits neuronal PANoptosis-like cell death by activating the Fxyd5/Lgals1 pathway

**Fig. 6.**
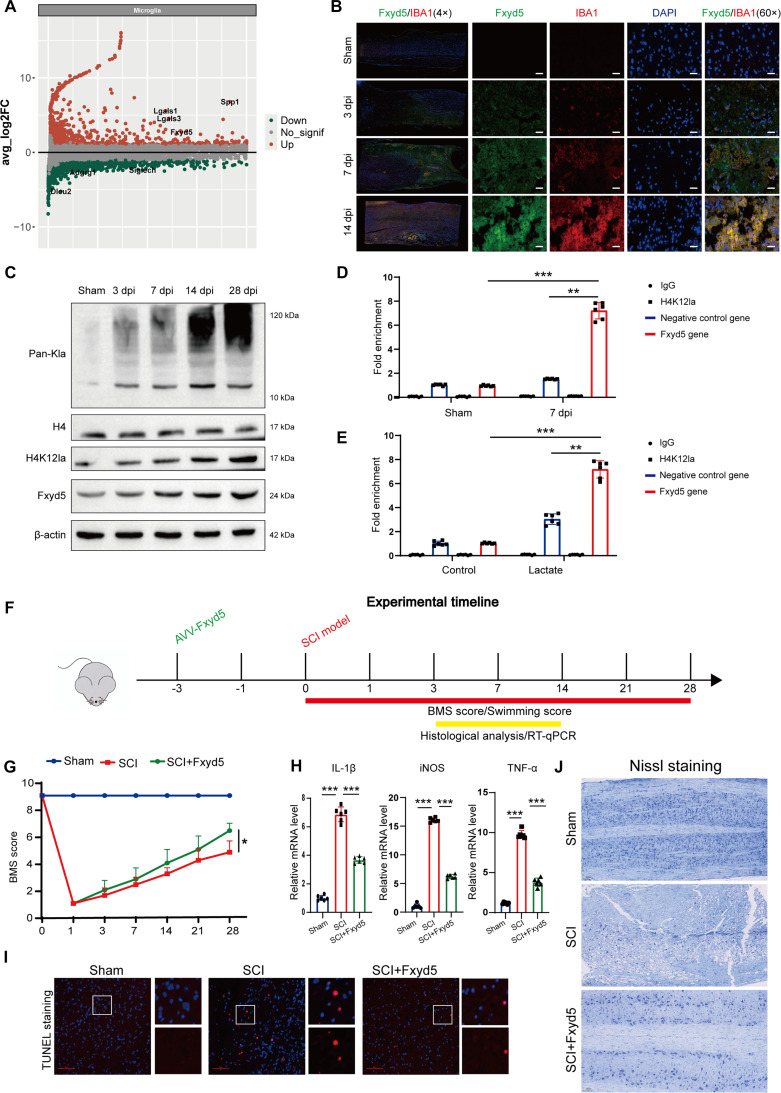
Lactate improves the outcomes of SCI by modulating the lactylation levels of Fxyd5. (A) A volcano plot illustrating differentially expressed genes in the microglia, with red indicating marked up-regulation, green for marked down-regulation, and gray for nonsignificant changes. (B) Relative immunofluorescence intensity of Fxyd5 in the microglial cells (scale bar = 20 μm). (C) Western blot analysis showing lactylation levels in the mouse spinal cords at various time points post-SCI. (D) ChIP assay detecting enrichment of H4K12la at the Fxyd5 promoter region following SCI. (E) ChIP assay examining the H4K12la enrichment at the Fxyd5 promoter region after lactate treatment. (F) Schematic diagram outlining the experimental procedures used in the study involving a mouse model. (G) Basso Mouse Scale (BMS) scores of mice at different time points posttreatment. (H) QPCR results assessing the changes in pro-inflammatory phenotypes of microglial cells following Fxyd5 overexpression. (I) TUNEL staining and quantification of TUNEL-positive cells (scale bar = 100 μm). (J) Nissl staining and quantification of Nissl-positive cells (scale bar = 250 μm). *n* = 6; data are presented as mean ± SEM; *P < 0.05; **P < 0.01; ***P < 0.001.

To deepen our understanding of how the microglia contribute to SCI repair, we analyzed the differentially expressed genes and identified a marked up-regulation of Fxyd5 (Fig. 6A and Fig. S9A and B). The IF analysis of the spinal cord tissues at various postinjury time points revealed that Fxyd5 is localized within the microglial cells, with its expression increasing over time after injury (Fig. 6B). Lactate enhances histone H4 lysine 12 lactylation following SCI, promoting microglial scar formation and functional recovery [15]. Our Western blot analysis confirmed that both the overall lactylation levels and H4K12la were markedly elevated in the 14- and 28-dpi groups compared to those of the 3- and 7-dpi groups (Fig. 6C). To explore whether histone lactylation regulates Fxyd5 transcription, chromatin immunoprecipitation (ChIP)-qPCR was performed using a nonregulatory intronic region of Gapdh as a negative control locus, with data normalized to input DNA (fold enrichment method). The results revealed enriched H4K12la at the Fxyd5 promoter region both after SCI and lactate treatment (Fig. 6D and E).

To explore the involvement of Fxyd5 in functional restoration and the regulation of the inflammatory environment following SCI, mice were allocated into 3 experimental groups: sham-operated, SCI, and SCI treated with AAV-Fxyd5 (Fig. 6F). Motor function evaluations revealed that the SCI + AAV-Fxyd5 group exhibited markedly higher BMS scores compared to the SCI group at 14, 21, and 28 dpi (Fig. 6G). Additionally, mRNA extraction from the spinal cord tissues containing the lesion epicenter demonstrated that Fxyd5 markedly reduced the SCI-induced elevation in IL-1β, iNOS, and TNF-α mRNA levels (Fig. 6H). TUNEL staining revealed fewer apoptotic cells in the SCI + AAV-Fxyd5 group than in the SCI group (Fig. 6I), whereas Nissl staining confirmed a protective effect on the neurons (Fig. 6J).

**Fig. 7.**
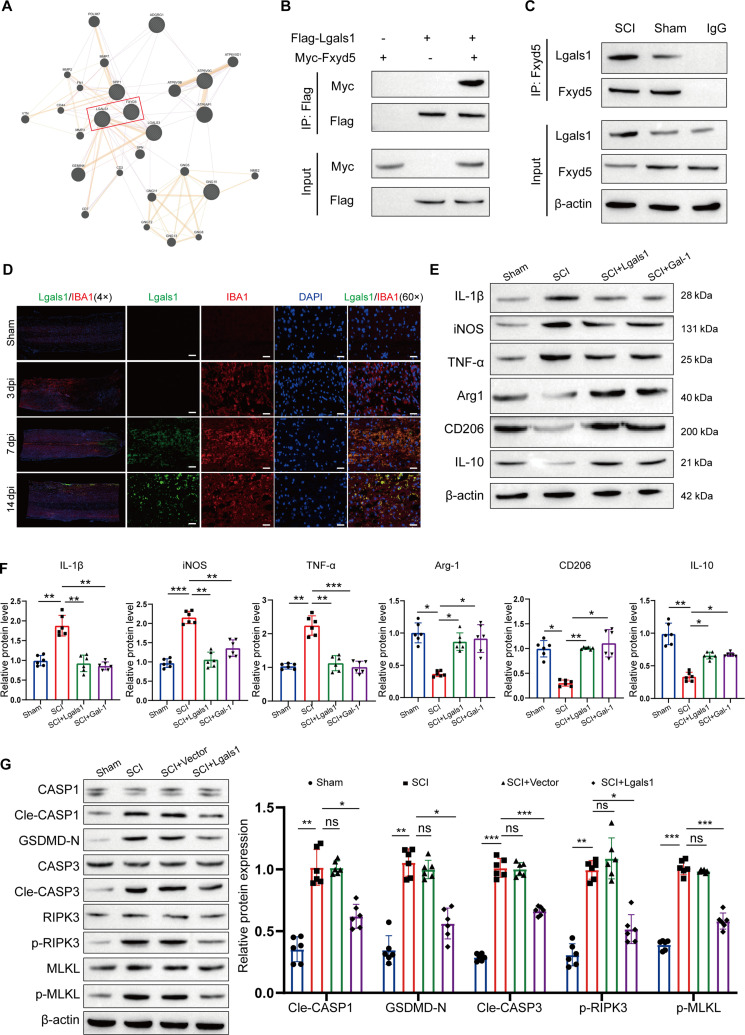
Microglial activation of the Fxyd5/Lgals1 pathway inhibits neuronal PANoptosis-like cell death. (A) PPI network analysis of differentially expressed genes in the microglia using the GeneMANIA database. (B and C) Pull-down and co-immunoprecipitation (co-IP) assays to detect the interaction between Fxyd5 and Lgals1. (D) Immunofluorescence analysis of Lgals1 expression in microglial cells (scale bar = 20 μm). (E and F) Western blot results showing the IL-1β, iNOS, TNF-α, Arg-1, CD206, and IL-10 levels. (G) Western blot analysis and quantification of proteins associated with PANoptosis-like cell death in the primary neuronal cells treated with Gal-1. Data are presented as mean ± SEM; *P < 0.05; **P < 0.01; ***P < 0.001. ns, not significant.

Further exploration using the GeneMANIA database for the PPI network analysis identified an interaction between Fxyd5 and Lgals1 (Fig. 7A). This interaction was validated through co-IP and pull-down assays (Fig. 7B and C). The glycan-dependent binding of galectin-1 and neuropeptide-1 can promote axonal regeneration after SCI [16]. The IF analysis also revealed that Lgals1 is up-regulated in microglial cells over time post-SCI (Fig. 7D). The presence of Lgals1 or exogenous Gal-1 decreased the pro-inflammatory markers while increasing the anti-inflammatory markers, indicating a shift toward an anti-inflammatory phenotype (Fig. 7E and F). Moreover, Lgals1 inhibited the SCI-induced neuronal apoptosis in the primary neuron cultures (Fig. 7G).

Primary microglia were isolated from wild-type mice, transduced with lentivirus to overexpress Fxyd5, and then transfected with Lgals1-specific siRNA. Results showed that Fxyd5 overexpression alone markedly up-regulated anti-inflammatory markers (e.g., Arg1, Cd206, and IL-10) and suppressed pro-inflammatory cytokines (e.g., IL-1β and TNF-α). However, upon Lgals1 knockdown, these beneficial effects of Fxyd5 were completely abolished, demonstrating that Lgals1 is indispensable for FXYD5-mediated anti-inflammatory reprogramming (Fig. S10A and B). In a microglia–neuron coculture system, microglia overexpressing Fxyd5 markedly enhanced neurite outgrowth and survival of primary cortical neurons. This neurosupportive effect was lost when Lgals1 was silenced in microglia, indicating that Lgals1 mediates the indirect neuroprotective function of FXYD5 (Fig. S10C to E). Adding recombinant LGALS1 protein to the culture medium of Lgals1-deficient microglia partially restored both anti-inflammatory gene expression and neuronal neurite growth, further confirming its functional role (Fig. S10C to E).

These findings collectively suggest that the up-regulation of histone lactylation in the microglial cells inhibits neuronal cell PANoptosis-like by activating the Fxyd5/Lgals1 pathway, offering fresh perspectives on possible therapeutic targets for SCI.

### GPR37L1-PSAP and GPR37-PSAP signaling identified through single-cell communication analysis

To identify additional pathways that may modulate the immune microenvironment following SCI, we performed an exploratory analysis of ligand–receptor interactions using a comprehensive database to compute the communication scores among various immune cell types in both sham and SCI conditions (Fig. S11A to D). Despite the many ligands expressed by microglia signaling to receptors on macrophages and astrocytes, the microglia–macrophage interactions involve more ligand–receptor pairs than the microglia–astrocyte interactions (Fig. S12A and B). These unique microglia–macrophage interactions include signaling related to GPR37L1-PSAP and GPR37-PSAP (Fig. S13). Overall, the chemotactic and inflammatory microglial subtypes exhibit similar interaction patterns with astrocytes and macrophages. Through overexpression of FKBP5 in microglia, we observed that FKBP5 up-regulates GPR37 at both the mRNA and protein levels (Fig. S14A and B). ChIP-qPCR assays further showed enhanced H4K12 lactylation at the GPR37 promoter upon FKBP5 overexpression (Fig. S14C), suggesting possible epigenetic regulation of GPR37 expression. However, it is important to note that these data demonstrate transcriptional up-regulation of GPR37, not functional activation of GPR37 signaling (e.g., ligand binding, receptor activation, or downstream phosphorylation cascades). We also found that co-knockdown of GPR37 in FKBP5-overexpressing microglia reversed certain FKBP5-induced phenotypes, including anti-inflammatory IL-10 secretion and promotion of neurite outgrowth (Fig. S14D and E). Nevertheless, given that GPR37 is canonically a receptor for extracellular ligands (PSAP) whose primary function is signal reception rather than downstream transcriptional output driven by intracellular lactate, these findings remain exploratory and await functional validation of the GPR37/PSAP signaling axis.

**Fig. 8.**
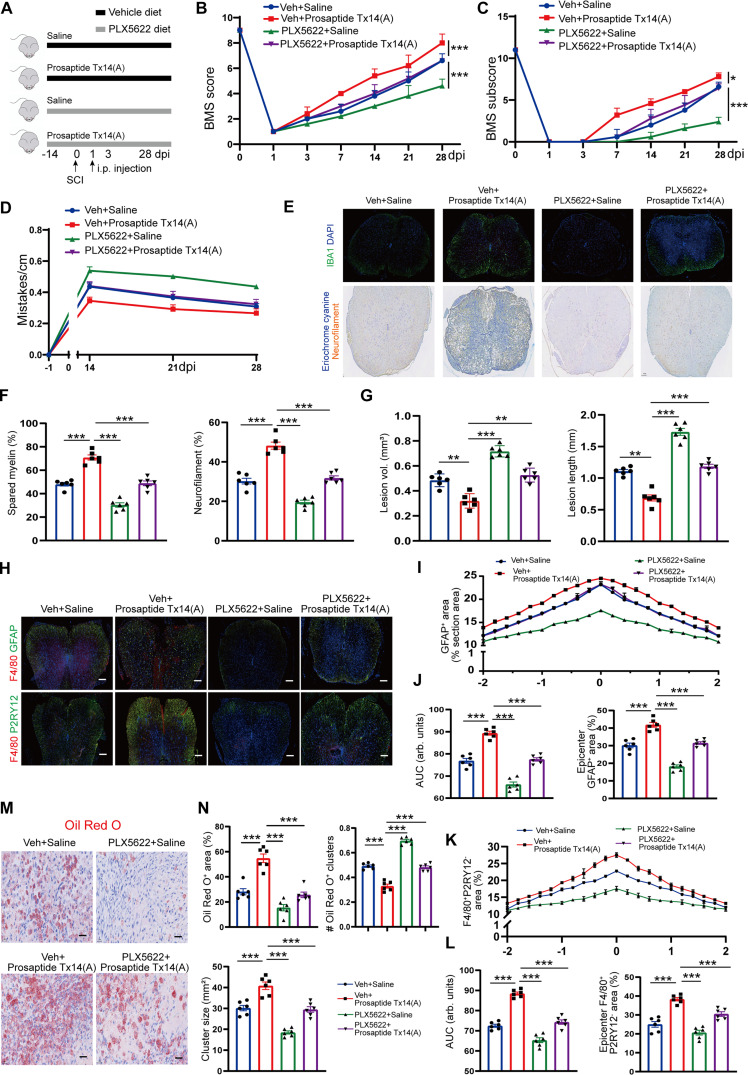
GPR37L1-PSAP and GPR37-PSAP signaling identified through single-cell communication analysis. (A) Mice were divided into different treatment groups following this timeline: starting 2 weeks before the spinal cord injury (SCI), mice were continuously fed either control chow or PLX5622 (PLX). At 1 dpi, Prosaptide TX14(A), a potent agonist of GPR37L1 and GPR37, was microinjected intraspinally (or saline was used as a control). At 3 dpi, Tlr2 agonist (Tlr2 ag) was administered intraperitoneally (or PBS as a control). Behavioral monitoring was continued until tissue collection at 28 dpi. (B to D) Based on the BMS scores (B), subscores (C), and horizontal ladder test results (D), the depletion of microglia markedly worsened the recovery after SCI. The administration of Prosaptide TX14(A) improved the motor function in the microglia-depleted mice but exacerbated the outcomes in mice with intact microglia. (E to G) In the microglia-depleted mice, reprogramming the macrophage/dendritic cell (MDM) phenotype enhanced tissue preservation; however, this effect was detrimental in the control mice (scale bar = 100 μm). (H to L) Prosaptide TX14(A) rescued glial scarring and MDM pathology in the microglia-depleted mice (scale bar = 100 μm). (M and N) Oil Red O staining indicated that Prosaptide TX14(A) improved lipid phagocytosis in the microglia-depleted mice (scale bar = 25 μm). Data are presented as mean ± SEM; *P < 0.05; **P < 0.01; ***P < 0.001. AUC, area under the curve.

In order to further explore the potential biological relevance of these ligand–receptor interactions, we depleted the microglia in mice using food laced with a CSF1R antagonist (PLX5622), starting 2 weeks before SCI and continuing until 28 dpi (Fig. S15A). All mice displayed a normal locomotion before SCI but developed near-complete paralysis at 1 dpi. Functional recovery was impaired in the microglia-depleted mice, which was associated with an exacerbated myelin and axonal pathology (Fig. S15B to P). Considering that Prosaptide TX14(A) is a potent agonist of GPR37L1 and GPR37, we hypothesized that injecting Prosaptide TX14(A) into the injury site could reverse the effects of microglial depletion. In a separate replicate experiment where survival was extended to 28 dpi (Fig. 8A), administration of Prosaptide TX14(A) mitigated the worsened pathological changes and functional deficits associated with microglial depletion (Fig. 8B to L).

Overall, these exploratory findings indicate that GPR37L1-PSAP and GPR37-PSAP signaling, identified through single-cell communication analysis, may play a role in regulating repair processes associated with neuroinflammation and reactive astrogliosis after SCI. However, unlike the mechanistically validated FKBP5–GPR84/IL-1β axis (acute phase) and FKBP5–LDHA–lactylation–FXYD5/LGALS1 axis (subacute phase), the GPR37/PSAP pathway has not been functionally linked to the FKBP5–lactylation axis in this study and is presented as an independent exploratory observation requiring future validation.

## Discussion

**Fig. 9.**
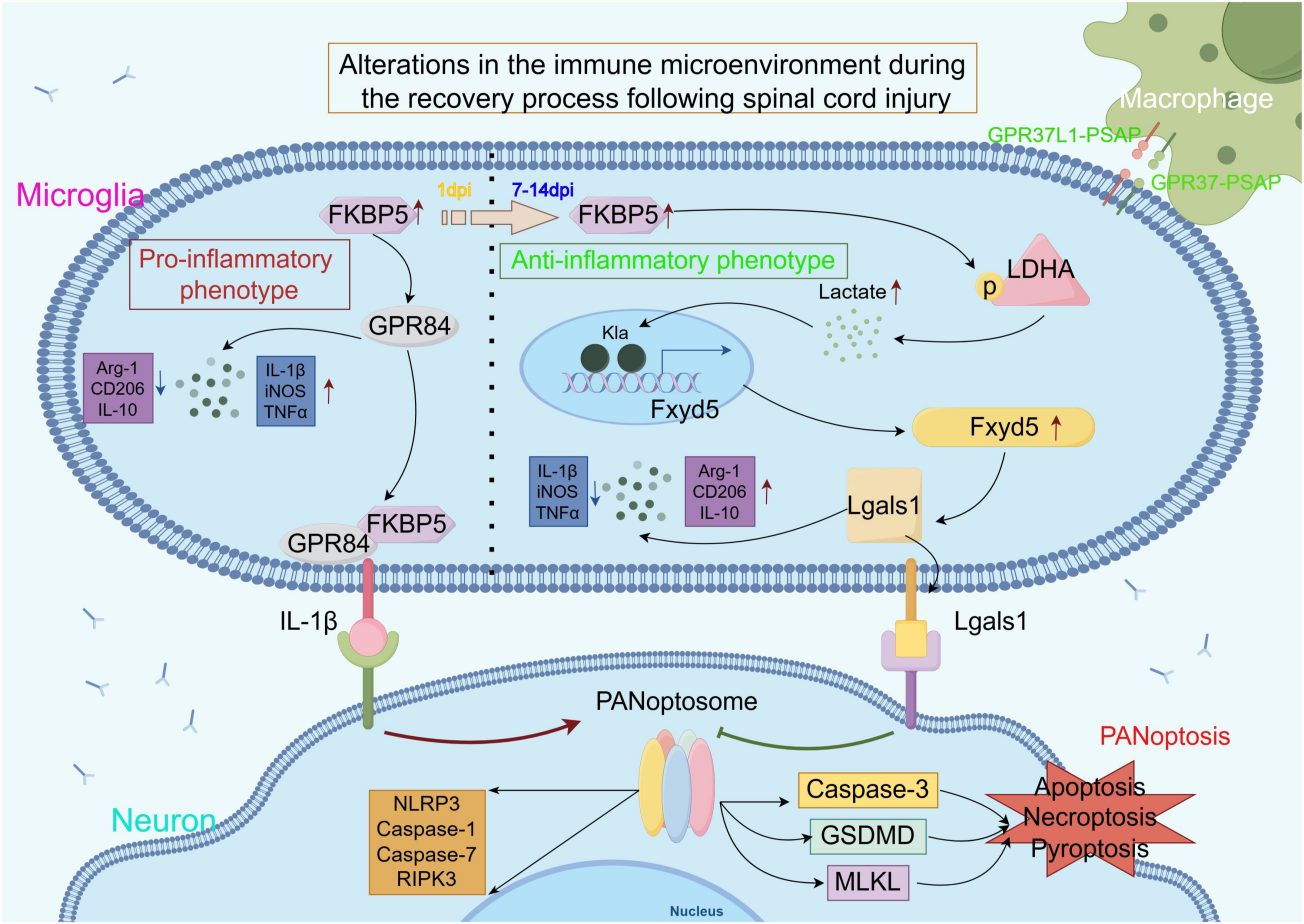
Mechanism pathway diagram. Following spinal cord injury, FKBP5 up-regulation initially activates the GPR84/IL-1β pathway, promoting microglial pro-inflammatory polarization and neuronal PANoptosis-like cell death. As FKBP5 levels increase, it induces metabolic reprogramming in microglia via LDHA phosphorylation, enhancing histone H4K12 lactylation at the Fxyd5 promoter to activate the FXYD5/LGALS1 pathway, thereby shifting microglia to an anti-inflammatory state and facilitating tissue repair.

SCI triggers a complex cascade of cellular and molecular events, with microglia playing a central role in both the initial inflammatory response and subsequent repair processes. The present study elucidates the dynamic roles of microglia in SCI pathology and recovery, highlighting their contributions to neuronal death, metabolic reprogramming, and immune modulation. Our findings reveal that microglia exhibit a biphasic response post-SCI—an early pro-inflammatory phase characterized by the induction of neuronal PANoptosis-like cell death, and a later reparative phase driven by metabolic and epigenetic reprogramming (Fig. 9). These observations offer a foundation for developing temporally precise therapeutic strategies aimed at mitigating secondary injury and enhancing functional restoration.

In the acute phase of SCI, the microglia rapidly transition into a pro-inflammatory phenotype, marked by the up-regulation of activation markers (e.g., SPP1 and CD68) and loss of homeostatic markers (e.g., P2Y12) [17,18]. This shift is accompanied by morphological changes from ramified to amoeboid shapes, consistent with their role in initiating the inflammatory response. We identified FKBP5 as a key regulator of this process, with its expression markedly elevated in microglia at 1 dpi. FKBP5, in collaboration with GPR84, amplifies IL-1β secretion, driving coordinated activation of pyroptosis, apoptosis, and necroptosis pathways—a phenomenon we cautiously describe as “PANoptosis-like” cell death. While our combinatorial inhibitor experiments (VX-765 + Z-VAD-FMK + Nec-1s) provide robust evidence for multipathway involvement and the co-IP data confirm physical interactions among RIPK3, Caspase-8, and NLRP3, we acknowledge that definitive demonstration of the canonical PANoptosome complex (e.g., involving ZBP1 and cFLIP) requires further investigation. Nevertheless, inhibition of FKBP5 or GPR84 attenuates this coordinated cell death, highlighting the therapeutic promise of modulating this pathway to mitigate neuronal damage during the initial phase of SCI. Notably, such context-dependent modulation of inflammatory signaling—where early blockade limits damage while preserving later repair functions—mirrors strategies emerging in cancer immunotherapy, where fine-tuning immune activation timing is critical for efficacy without collateral toxicity [19].

As the injury progresses, microglia undergo metabolic reprogramming [15,20]. This transition is mediated by FKBP5, which directly interacts with LDHA to regulate its phosphorylation at Tyr10, enhancing lactate production. Lactate, once considered a metabolic waste product, emerges as a critical signaling molecule that promotes histone lactylation, particularly at the H4K12 site. Through ChIP-qPCR assays with appropriate genomic control loci (including a nonregulatory intronic region of Gapdh as a negative control) and normalization to input DNA, we demonstrated that this epigenetic modification activates the transcription of Fxyd5, a gene implicated in anti-inflammatory responses. The up-regulation of FXYD5 and its downstream effector LGALS1 (galectin-1) shifts microglia toward an anti-inflammatory state, characterized by reduced expression of pro-inflammatory markers (e.g., IL-1β, iNOS, and TNF-α) and increased expression of anti-inflammatory markers (e.g., Arg-1, CD206, and IL-10). This metabolic–epigenetic axis not only suppresses neuronal PANoptosis-like death but also promotes tissue repair and functional recovery, as evidenced by improved BMS scores and reduced neuronal apoptosis in lactate-treated SCI mice. The concept that metabolic intermediates like lactate serve as substrates for histone modifications to direct gene expression aligns with recent findings in oncology, where lactylation-driven transcriptional programs govern cell fate decisions through RNA-binding proteins and epitranscriptomic regulators [21], reinforcing the generality of metabolism–epigenetics coupling across pathophysiological contexts.

While our work demonstrates that FKBP5-driven lactate production enhances H4K12 lactylation at the Fxyd5 promoter to promote microglial repair functions, it is increasingly recognized that lysine lactylation also modifies numerous nonhistone proteins—such as metabolic enzymes (e.g., GAPDH and PKM2), cytoskeletal components, and signaling molecules—thereby influencing diverse cellular processes. Given that FKBP5 modulates intracellular lactate levels by regulating LDHA activity, it is plausible that FKBP5 indirectly shapes the global lactylome in activated microglia. For instance, lactylation of key glycolytic or inflammatory regulators could further amplify or fine-tune the phenotypic switch. Although our current study focuses on the epigenetic regulation of Fxyd5, future proteome-wide lactylome profiling (e.g., using anti-Kla immunoprecipitation coupled with mass spectrometry) in Fkbp5-deficient microglia would be instrumental in defining the full scope of FKBP5’s influence on protein lactylation networks.

Beyond their intrinsic roles, microglia orchestrate the immune microenvironment by interacting with other immune cells, particularly macrophages [22,23]. Through single-cell ligand–receptor communication analysis, we identified enriched interactions between microglia and macrophages, including the GPR37L1–PSAP and GPR37–PSAP pathways. However, it is important to clarify the distinction between our core mechanistic findings and these exploratory observations. While FKBP5 overexpression up-regulates GPR37 mRNA and protein levels and enhances H4K12la enrichment at its promoter, these data only suggest possible epigenetic regulation of GPR37 expression. They do not demonstrate that GPR37 signaling activity—including ligand binding, receptor activation, or downstream phosphorylation cascades—is directly regulated by the FKBP5–lactate axis. Given that GPR37 is canonically a receptor for extracellular ligands (PSAP) whose primary function is signal reception rather than downstream transcriptional output driven by intracellular lactate, we present these findings strictly as exploratory observations that warrant independent functional validation. Depletion of microglia disrupts the containment of monocyte-derived macrophages (MDMs) within the lesion core, leading to ectopic infiltration and exacerbated demyelination, while administration of Prosaptide TX14(A), a GPR37L1/37 agonist, restores glial scar integrity and MDM corralling. These results highlight the importance of microglia-dependent signaling in coordinating immune responses post-SCI, but the functional connection between GPR37/PSAP signaling and the FKBP5–LDHA–lactylation–FXYD5 axis requires future investigation. Interestingly, the dual-phase nature of inflammation—destructive early but reparative late—is also observed in nonneural tissues such as the kidney, where IL-1 family cytokines initially drive injury but later contribute to resolution and fibrosis control [24]. This cross-organ conservation underscores the evolutionary importance of temporally regulated immune responses and cautions against blanket anti-inflammatory interventions.

The dual roles of the microglia in SCI—both as drivers of secondary damage and as facilitators of repair—underscore the need for temporally precise interventions [25,26]. Early targeting of FKBP5/GPR84/IL-1β signaling may mitigate the neuronal PANoptosis-like cell death, whereas later modulation of lactate metabolism and histone lactylation could promote anti-inflammatory and reparative microglial phenotypes. Additionally, enhancing the microglia–macrophage crosstalk through the GPR37L1/37 agonists offers a promising avenue for optimizing the immune microenvironment. However, the context-dependent effects of these interventions emphasize the importance of tailored therapeutic strategies.

This study demonstrates that the primary role of FKBP5 in SCI repair is to drive microglial phenotypic transition via a metabolic–epigenetic–signaling cascade. Specifically, the LDHA–lactic acid–H4K12 lactoylation–FXYD5/LGALS1 axis represents a critical pathway during the subacute phase of SCI repair, with its causal relationship substantiated through multiple experimental approaches, including ChIP-qPCR, AAV-mediated interventions, and lactic acid supplementation. In contrast, the GPR84/IL-1β pathway plays a dominant role in amplifying inflammatory responses during the acute phase. Other interactions identified through single-cell communication analyses, such as GPR37L1–PSAP, remain to be functionally validated and may constitute auxiliary regulatory networks within the injury microenvironment, warranting further investigation. Although GPR37/GPR37L1 has not been directly linked to FKBP5 or lactylation in our current study, its marked up-regulation in reparative-phase microglia suggests that it may function as a potential sensor in the lactate-enriched microenvironment. Given prior reports that GPR37 responds to lipid metabolites and modulates neuroinflammation, we speculate that lactate or lactylation might indirectly influence its activity—a hypothesis warranting future investigation.

While our study focuses on SCI, the FKBP5–LDHA–lactylation–FXYD5 axis may represent a more general mechanism by which microglia integrate metabolic and epigenetic signals to modulate neuroinflammation and repair. Notably, lactate accumulation, microglial activation, and histone lactylation have also been reported in other CNS insults, including traumatic brain injury (TBI) and ischemic stroke. For instance, elevated lactate levels are a hallmark of the penumbra in stroke and correlate with microglial reactivity; similarly, TBI induces rapid metabolic shifts and inflammatory responses that share features with SCI. Given that FKBP5 is stress-inducible and LDHA is ubiquitously expressed in activated glia, it is plausible that this metabolic–epigenetic axis operates across multiple CNS injury contexts. Future studies in TBI, stroke, or neurodegenerative models will be essential to determine whether targeting this pathway could offer broad therapeutic benefits beyond SCI.

The observation that FKBP5 promotes inflammation via the GPR84/IL-1β pathway in the acute phase and mediates repair through LDHA-dependent metabolic reprogramming in the later phase raises a critical question: How does a single molecule orchestrate such contrasting functions at different stages postinjury? Several mechanisms likely underlie this temporal shift. Firstly, FKBP5 expression is dynamically regulated by stress signals following SCI, with up-regulated levels during the acute phase enhancing pro-inflammatory pathways like GPR84/IL-1β through direct interactions or modulation of glucocorticoid receptor activity. As the injury environment evolves, changes in local cytokine profiles and cellular metabolism alter FKBP5’s functional outcomes. Secondly, posttranslational modifications (PTMs) such as phosphorylation, acetylation, and ubiquitination modify FKBP5’s stability, localization, and interaction partners, potentially differing between acute and reparative phases. For instance, phosphorylation may promote its association with GR in the acute phase, while dephosphorylation facilitates interaction with LDHA during repair. Thirdly, the cellular milieu surrounding FKBP5-expressing cells dramatically changes over time, shifting from a pro-inflammatory environment dominated by activated microglia and immune cells to an anti-inflammatory state conducive to tissue repair. Lastly, feedback loops and crosstalk between inflammatory and metabolic pathways contribute to FKBP5’s dual roles, with early inflammatory responses influencing metabolic reprogramming and vice versa. Further longitudinal studies tracking FKBP5 expression and PTMs, along with detailed analyses of its interacting partners, are essential for understanding how FKBP5 coordinates both inflammatory and reparative processes in response to SCI.

In summary, the present study offers a comprehensive understanding of the multifaceted roles of microglia in SCI, with a clearly delineated core mechanism (FKBP5–GPR84/IL-1β in the acute phase; FKBP5–LDHA–lactylation–FXYD5/LGALS1 in the subacute phase) and several exploratory observations (GPR37/PSAP signaling) that provide directions for future research. By elucidating the molecular mechanisms underlying microglial activation, metabolic reprogramming, and immune modulation, we have identified several potential therapeutic targets for improving outcomes after SCI. Future studies should explore the translational potential of these findings, with a focus on developing temporally and spatially precise interventions that harness the reparative capacity of microglia while minimizing their detrimental effects. We also acknowledge that our conclusions regarding PANoptosis are based on pharmacological inhibition and co-IP evidence of multipathway activation rather than definitive demonstration of the complete PANoptosome complex; therefore, we describe this phenomenon as “PANoptosis-like” cell death. Future investigations directly examining ZBP1, cFLIP, and other core PANoptosome components will be essential to fully establish the molecular architecture of this cell death pathway in the context of SCI.

## Conclusion

The present study offers a comprehensive understanding of the multifaceted roles of microglia in SCI. By elucidating the molecular mechanisms underlying microglial activation, metabolic reprogramming, and immune modulation, we have identified several potential therapeutic targets for improving the outcomes after SCI. In the future, studies should explore the translational potential of these findings, with a focus on developing temporally and spatially precise interventions to harness the reparative capacity of the microglia while minimizing their detrimental effects.

## Methods

### Animals

Female animals are commonly selected for experimental studies on SCI because their shorter urethras offer a practical advantage, making manual bladder expression more straightforward and reducing the risk of urinary retention after injury compared to males. In this investigation, 6-week-old female C57BL/6 mice, weighing 20 to 30 g, were obtained from the Experimental Animal Center of Kunming Medical University. The animals were maintained in standard laboratory housing conditions with a regulated 12-h light–dark cycle and provided food and water ad libitum. All experimental protocols involving animals were carried out in accordance with established ethical standards and received prior approval from the Animal Care and Use Commission of Kunming Medical University.

### Animal model of SCI

Before experimentation, mice were anesthetized by intraperitoneal administration of 1% (w/v) sodium pentobarbital at a dose of 50 mg/kg. A laminectomy was carried out at the T9 to T10 spinal segment to expose a circular area of the dura mater. SCI was produced using a standardized weight-drop technique as described earlier [9]. In detail, a 10-g rod, 1.2 mm in diameter, was released from a height of 15 mm onto the exposed spinal cord to generate a moderate contusion injury, taking care to preserve the integrity of the dura. After the injury induction, the muscle layers, fascia, and skin were closed with 4-0 nonabsorbable silk sutures. Animals in the sham group underwent identical surgical procedures except for the absence of the weight impact. Postoperatively, bladder expression was performed manually 3 times per day to prevent urinary retention.

### Single-cell analysis methods

For single-cell analysis, we selected the GSE162610 dataset, which comprises single-cell data from mouse spinal cord tissues. This dataset includes a total of 10 samples: 3 from untreated (noninjured) conditions and 7 from SCI conditions. To ensure high-quality cells for annotation, we excluded cells with nFeature_RNA < 200, nFeature_RNA > 6,000, nCount_RNA < 200, or nCount_RNA > 30,000 using the Seurat package in R.

For cell clustering, dimensionality reduction and clustering were performed using the Seurat package in R. The dimension (Dim) was set to 16 by selecting dimensions with a marked drop in variance. “Harmony” was employed for batch correction, setting the single-cell resolution to 0.1. Cell clustering visualization was conducted using the UMAP method. Marker genes used for cell annotation were sourced from literature [27].

Differential expression analysis for single-cell data was performed by selecting genes with an absolute fold change (|FC|) greater than 2 and a *P* value less than 0.05 as differentially expressed genes. For analyzing cell–cell communication, the “cellchat” package in R was utilized.

### Statistics

All statistical evaluations were performed with GraphPad software under double-blind conditions. Results are expressed as mean values accompanied by the standard error of the mean (SEM). To reduce experimental variability, data normalization procedures were implemented. Independent 2-group comparisons were analyzed using a 2-tailed unpaired *t* test. When comparing 3 or more groups involving normally distributed datasets, 2-way analysis of variance was conducted, followed by Tukey’s post hoc test for multiple comparisons. In cases where data did not follow a normal distribution, the nonparametric Mann–Whitney *U* test was applied. Statistical significance was defined as a *P* value below 0.05.

Other experimental methods are described in the Supplementary Materials.

## Ethical Approval

This study and included experimental procedures were approved by The First Affiliated Hospital of Kunming Medical University. All animal experiments were approved by the Institutional Animal Care and Use Commission of Kunming Medical University (Approval No. KMMUD2026011).

## Data Availability

The datasets used and/or analyzed during the current study are available from the corresponding author on reasonable request.
