# Supplementary material for: FKBP5 Orchestrates a Biphasic Microglial Response in Spinal Cord Injury by Sequentially Activating the GPR84/IL-1β Pathway and the LDHA–Lactylation–FXYD5/LGALS1 Axis
Source: Research (Wash D C). 2026 Aug 4;9:1384. doi: 10.34133/research.1384 (PMC13433926; doi:10.34133/research.1384)
Supplement: Supplementary file 1 [file research.1384.f1.zip › Supplementary.docx]

**Reagents:**
The primary antibodies and reagents listed below were utilized in

this study and obtained from the indicated source: diaminobenzidine (DAB), haematoxylin and eosin (HE) dyes, Masson staining reagent, pentobarbital sodium, and Nissl staining reagent were purchased from Solarbio Science & Technology (PRC). Cytoplasmic Extraction Reagent and NE-PER™ Nuclear and bicinchoninic acid (BCA) reagents were purchased from Thermo Fisher Scientific (USA). A horseradish peroxidase (HRP)-conjugated IgG secondary antibody was obtained from Santa Cruz Biotechnology (USA). Compound C (CC, C24H25N5O; purity ≥98.14%) was obtained from MedChemExpress (USA; Cat# HY13418A). 4′,6-Diamidino-2-phenylindole (DAPI) was obtained from Beyotime Biotechnology (PRC).

**Antibodies**:

***Primary Antibodies***

| **Target** | **Host/Clonality** | **Vendor** | **Catalog No.** | **Application & Dilution** |
| --- | --- | --- | --- | --- |
| FKBP5 | Rabbit polyclonal | Abcam | ab126715 | WB 1:1000; IF 1:200 |
| LDHA | Rabbit monoclonal | Abcam | ab52488 | WB 1:1000 |
| p-LDHA (Y10) | Rabbit monoclonal | Cell Signaling Technology | #19A7 (RRID: AB_2892065) | WB 1:1000 |
| H4K12la | Rabbit polyclonal | PTM Biolabs | PTM-701 | WB 1:1000; IF 1:200 |
| Histone H4 | Rabbit monoclonal | Proteintech | 83095-1-RR | WB 1:2000 |
| FXYD5 | Rabbit polyclonal | Proteintech | 12166-1-AP | WB 1:1000; IF 1:100 |
| LGALS1 | Rabbit monoclonal | Cell Signaling Technology | #12936 | WB 1:1000; IF 1:100 |
| GPR84 | Rabbit monoclonal | Cell Signaling Technology | #61197 | WB 1:1000 |
| IL-1β | Rabbit polyclonal | ABclonal | A1112 | WB 1:1000; IF 1:200 |
| Caspase-1 | Rabbit polyclonal | Proteintech | 22915-1 | WB 1:1000 |
| Cleaved Caspase-8 | Rabbit monoclonal | Cell Signaling Technology | #97756 | WB 1:1000 |
| RIPK1 | Rabbit polyclonal | Abcam | ab62344 | WB 1:1000 |
| RIPK3 | Rabbit polyclonal | Abcam | ab180799 | WB 1:1000 |
| MLKL | Rabbit polyclonal | Affinity Biosciences | DF7412 | WB 1:1000 |
| GSDMD | Rabbit polyclonal | Affinity Biosciences | AF4013 | WB 1:1000 |
| IBA1 | Rabbit polyclonal | Abcam | ab178846 | IF 1:500 |
| NeuN | Mouse monoclonal | Abcam | ab56416 | IF 1:500 |
| MAP2 | Chicken polyclonal | Abcam | ab104224 | IF 1:500 |
| Synaptophysin | Mouse monoclonal | Abcam | ab183830 | IF 1:200 |
| GFAP | Mouse monoclonal | Thermo Fisher | 13-0300 | IF 1:500 |
| Arg-1 | Rabbit monoclonal | Cell Signaling Technology | #93668 | IF 1:200 |
| CD206 | Rabbit monoclonal | Cell Signaling Technology | #24595 | IF 1:200 |
| iNOS | Rabbit monoclonal | Abcam | ab178945 | IF 1:200 |
| TNF-α | Rabbit monoclonal | Abcam | ab183218 | IF 1:200 |
| IL-10 | Rabbit polyclonal | Abcam | ab189392 | IF 1:200 |
| SPP1 (Osteopontin) | Goat polyclonal | R&D Systems | AF808 | IF 1:100 |
| Ly6G/Ly6C | Rat monoclonal | R&D Systems | MAB1037 | IF 1:200 |
| F4/80 | Rat monoclonal | Abcam | ab300421 | IF 1:200 |
| CD68 | Rat monoclonal | Bio-Rad | MCA1957 | IF 1:200 |
| P2Y12 | Rabbit polyclonal | AnaSpec | AS-55043A | IF 1:200 |
| CSPG | Mouse monoclonal | Sigma-Aldrich | C8035 | IF 1:200 |
| Fibronectin | Rabbit polyclonal | Millipore | AB2033 | IF 1:200 |
| Collagen I | Rabbit polyclonal | Abcam | ab21286 | IF 1:200 |
| 5-HT | Rabbit polyclonal | Immunostar | 20080/20079 | IF 1:1000 |
| FGFR1 | Rabbit monoclonal | Cell Signaling Technology | #9740 | WB 1:1000 |
| β-actin | Mouse monoclonal | Abcam | ab6276 | WB 1:5000 |

***Secondary Antibodies***

| **Secondary Antibody** | **Vendor** | **Catalog No.** | **Application & Dilution** |
| --- | --- | --- | --- |
| Goat anti-mouse IgG H&L (Alexa Fluor® 647) | Abcam | ab106393 | IF 1:500 |
| Goat anti-rabbit IgG H&L (Alexa Fluor® 488) | Abcam | ab150115 | IF 1:500 |
| Other fluorophore- or HRP-conjugated secondary antibodies (as applicable) are also listed in the same format. |  |  |  |

**Primary Microglial Culture**

Primary microglia were isolated from the brains of newborn mice (aged 0-3 days). After removing blood vessels and meninges, brain tissues were minced and digested with 0.25% trypsin (Biological Industries) for 25 minutes. Then, 3 ml of culture medium was added to terminate the digestion process. The cell suspension was gently pipetted up and down ten times using a 1-ml pipette, then filtered through a 40-μm mesh filter. It was subsequently centrifuged at 1200 rpm for 5 minutes to obtain a mixed glial cell population. The cells were seeded into poly-D-lysine-coated T25 flasks and maintained in DMEM/F12 medium containing fetal bovine serum (FBS; Thermo-Fisher Scientific), penicillin, and streptomycin. After a 14-day in vitro culture period, flasks were agitated at 250 rpm for 6 hours to detach mature microglia. Harvested microglia were plated in culture dishes and kept at 37°C in a humidified atmosphere with 5% CO2. Prior to each experiment, microglia were seeded at a density of approximately 1×10^6 cells/ml and allowed to adhere for 24 hours.

**Cell Culture and Primary Neuron Isolation**

HEK293T cells (Cat#: FH0244) were obtained from the National Collection of Authenticated Cell Cultures in Shanghai, China. These cells were cultured in Dulbecco’s Modified Eagle Medium (DMEM; Gibco, Waltham, MA), supplemented with 10% fetal bovine serum (FBS; Thermo-Fisher Scientific). Primary neurons were isolated by trypsin digestion. Briefly, finely chopped neural tissue was treated with 0.05% trypsin (Gibco, Waltham, MA) containing 20 U mL^-1 DNase (Beyotime, Shanghai, China) for a total digestion time of 12 minutes in a 37°C incubator, stirring every 4 minutes. Digestion was stopped by adding an equal volume of DMEM containing 10% FBS, and the supernatant was carefully transferred to a 15 mL centrifuge tube. Cells in the supernatant were counted and seeded onto cell culture dishes coated with poly-L-lysine (Beyotime, Shanghai, China). After 24 hours, the medium was replaced with neurobasal medium (Gibco, Waltham, MA) enriched with 2% B27 (Gibco, Waltham, MA) and 1% GlutaMAX-I (Gibco, Waltham, MA). Throughout the culture period, half of the medium was exchanged every 2 to 3 days.

**Preparation of Tissues and HE, Nissl, and Masson Staining**

On days 1, 3, 7, 14, and 28 post-SCI, mice were anesthetized with 2% (w/v) pentobarbital sodium and perfused with normal saline followed by 4% (w/v) paraformaldehyde (PFA) in PBS. A segment of the spinal cord rostral to the injury (1 mm long, 4 mm distant from the injury center) along with the entire injured section (10 mm in length, centered on the injury site) were excised and postfixed in 4% (w/v) PFA for one day. The tissues were dehydrated in a series of ethanol solutions of increasing concentrations. After clearing, specimens were embedded in paraffin. Sections of 4 μm thickness were cut and mounted onto gelatin-coated slides. Rostral spinal cord segments and the whole sections were sectioned into longitudinal and transverse slices, respectively. These slices were stained with H&E, and for Nissl staining, transverse sections were stained with 1% cresyl violet according to the manufacturer's guidelines12. Motor neurons within the anterior horns were identified as Nissl-positive cells based on prior methodologies13. For Masson staining, longitudinal sections were permeabilized using 10% K2Cr2O7 and 10% trichloroacetic acid (TCAA), and nuclei were stained with haematoxylin. Slides were then differentiated with HCl and ethyl alcohol, blued in dilute ammonia, and finally stained with Masson's trichrome14. Bright-field images of H&E, Masson, and Nissl staining were captured using a light microscope equipped with a 4× objective lens (Olympus, Japan). ImageJ software's thresholding method was utilized to quantify the blue-stained area in Masson-stained samples. The count of Nissl-positive motor neurons in the anterior horn region was manually determined in a double-blind manner.

**Quantification of IF Staining**

Samples stained for MAP2, Caspase-1, Caspase-3, and RIPK3 were imaged using a Zeiss LSM 800 confocal microscope. Image acquisition and processing were performed with Zen Blue software (Zeiss), while ImageJ software (Version 1.52a) was utilized for preparing images for presentation. Target regions were photographed at a resolution of 1024 × 1024 pixels using a 20× objective lens without employing optical zoom through confocal laser scanning microscopy. Specimens stained for MAP2, Caspase-1, Caspase-3, and RIPK3 were observed and captured with a fluorescence microscope equipped with a 10× objective lens (Olympus, Japan), ensuring that the exposure time remained consistent for each marker. For transverse sections, imaging was conducted 0.5–1 mm cranial to the injury site; six random areas within the anterior horn from three different sections per animal were selected for imaging. The integrated density of MAP2, Caspase-1, Caspase-3, and RIPK3 in neurons marked by NeuN was calculated using ImageJ software. The counts of SYN-positive synapses within neurons were manually quantified in a double-blind manner.

**Western Blot (WB) Analyses**

On the first and third days following spinal cord injury (SCI), mice were humanely euthanized, and spinal cord segments (1.5 cm in length, encompassing the injury site) were collected and stored at -80°C until ready for Western blot analysis. Proteins were extracted from some samples using lysis buffer. For the remaining specimens, nuclear and cytoplasmic proteins were isolated using Cytoplasm Extraction Reagent and NE-PER™ Nuclear Extraction Reagents, respectively. Total protein from the spinal cord samples was purified using specific extraction reagents. Protein concentration was determined through BCA assay. Equal quantities of protein (60 μg) were separated via 12% (w/v) SDS-PAGE. Proteins were then transferred onto PVDF membranes (Roche Applied Science, USA) and blocked with 5% (w/v) skim milk before being incubated overnight at 4°C with antibodies against various markers including FKBP5, GPR84, LDHA, Fxyd5, Lgals1, iNOS, TNF-α, Arg-1, CD206, histone-H4, IL-10, FGFR1, RIPK3, C-Caspase-3, C-Caspase-8, MLKL, Caspase-1, β-actin, GSDMD, and IL-1β, all at dilutions of 1:1000. After this, the membranes were incubated with HRP-conjugated secondary antibodies at room temperature for two hours. Finally, the bands were visualized using a ChemiDoc™ XRS+ imaging system (Bio-Rad) according to the instructions provided with an enhanced chemiluminescence detection kit.

**Immunoprecipitation**

The procedure for immunoprecipitation was carried out following previously outlined methods16. Spinal cord samples were homogenized in a buffer containing IGEPAL CA-630 (50 mM Tris-HCl, 1% IGEPAL CA-630, 10 mM EDTA, 150 mM NaCl, 50 mM NaF, 1 μM leupeptin, and 0.1 μM aprotinin). Post centrifugation, the supernatant of the homogenate was gathered for immunoprecipitation. Using the manufacturer’s protocol, the primary antibody was bound covalently to protein A/G agarose utilizing a Pierce Crosslink Immunoprecipitation Kit. The samples were then incubated with these immobilized antibody beads at 4°C for 2 hours. Additionally, an IgG isotype control was used for immunoprecipitation of the samples. After completing the immunoprecipitation process and washing with Tris-buffered saline (TBS), the samples were eluted using glycine-HCl (0.1 M, pH 3.5), and subsequently analyzed by Western blotting with specific primary antibodies against the proteins of interest.

**In vitro Pull-down Assay**

The Flag-tagged FKBP5 recombinant protein was expressed and purified from HEK293 cells. His-tagged LDHA was sourced from Abcam (product number ab93699, located in Cambridge, UK). To conduct His pull-down assays, HisPur Cobalt resin (Thermo Fisher Scientific, catalog number 21277, Massachusetts, USA) was utilized, incubating with FKBP5 proteins overnight at 4°C. For Flag pull-down experiments, they were executed using Flag-tagged FKBP5 proteins bound to anti-DYKDDDDK affinity resin (Thermo Fisher Scientific, product code A36803, also from Massachusetts, USA). Proteins that interacted were extracted using an imidazole-based elution buffer before undergoing SDS-PAGE for analysis.

**QPCR**

Total RNA was isolated from spinal cord tissues using TRIzol reagent. cDNA synthesis was performed with PrimeScript II 1st Strand cDNA Synthesis Reagent (6210B, Takara) following the manufacturer’s instructions. Primer sequences for target genes were designed based on GenBank data and synthesized by Nanjing Zoonbio Biological Technology Company. Real-time PCR was conducted using SYBR Premix Ex Taq (RR420A, TAKARA) under the following conditions: 30 cycles of denaturation at 95 °C for 0.5 min, annealing at 65 °C for 0.5 min, and extension at 72 °C for 45 s, followed by signal detection at 72 °C. Target mRNA expression levels were normalized to β-actin mRNA levels.

The sequences were as follows:

FKBP5 (forward: 5′-GGCAAACTCTAGGAGGCTTAAA-3′,

reverse: 5′- CTTACAGCTCCCACCAGAATAC-3′),

IL-1β (forward: 5′- TGGCAACTGTTCCTG-3′, reverse: 5′- GGAAGCAGCCCTTCATCTTT-3′), iNOS (forward: 5′- GCGTCCACCGTCTTCATT-3′, reverse: 5′- CCCTCGGCGATGCTG-3′), TNF-α (forward: 5′- CCAATGGCAGAGTGGGTATG-3′,

reverse: 5′- TGAAGAGGACCTGGGAGTAG-3′), and

Fxyd5 (forward: 5′- ATGTCACTGTCCAGTCGCCTGTGTCTCCTCACT-3′,

reverse: 5′- TCACCTGTGGCGATTCAGGCAAAATTGAGACAA-3′).

**Pharmacological Microglia Depletion**

A colony-stimulating factor 1 receptor (Csf1R) antagonist, PLX5622 (formulated at a concentration of 1200 mg/kg in chow; orally; 290 ppm, Plexxikon), was utilized to pharmacologically deplete microglia. PLX5622 is an orally bioavailable compound that can cross the blood-brain barrier and specifically inhibits Csf1R tyrosine kinase activity with a 50-fold greater selectivity compared to four related kinases19. For all experiments involving PLX5622, animals were first randomly assigned to cages, and then cages were randomly allocated to dietary groups using QuickCalcs (GraphPad software). All animals within the same cage received the identical diet.

**General Pathology**

Sections stained for endothelial cells/neurofilament were digitized using a ScanScope XT scanner (Aperio; OSU Comparative Pathology and Mouse Phenotyping Shared Resource; College of Veterinary Medicine) along with ImageScope software (Leica Biosystems). For lesion analysis, ImageJ was utilized to examine sections spaced 0.2 mm apart across a 4 mm span extending from 2 mm rostral to 2 mm caudal of the lesion epicenter. The damaged tissue within this area, identified by neurofilament staining, was outlined in ImageJ using the polygon selection tool. Lesion volume was calculated as the sum of (the area of the lesioned tissue per section multiplied by the distance between sections). Lesion length was determined as the rostral-caudal span covered by sections containing the lesioned tissue. Three-dimensional reconstructions of contusion SCI lesions were created using M3D from MCID Elite Software. In the case of contusion SCI, spared myelin and/or neurofilament were outlined in ImageJ with the polygon selection tool and expressed as a percentage of the section area. For crush SCI, the epicenter was defined as the horizontal section at the level of the central canal, and the spared myelin area was reported as a percentage of EC+ tissue within a 2 mm-length region centered on the crush site.

**Microglia**

For contusion SCI, sections spaced 0.2 mm apart, extending from 2 mm rostral to 2 mm caudal of the epicenter, were stained for P2RY12 and processed using immunoperoxidase staining. These sections were then digitized with a ScanScope XT scanner (Aperio) and ImageScope software (Leica Biosystems). In the case of contusion SCI, the count of P2RY12+ cells was performed using Imaris and reported as a percentage of the total section area, which was delineated in ImageJ. For crush SCI, the extent of microglial coverage was expressed as a percentage of P2RY12+ tissue within a 2 mm-length region centered on the crush site.

**MDMs**

For contusion SCI, spinal sections spaced 0.2 mm apart, extending from 2 mm rostral to 2 mm caudal of the epicenter, were stained for P2RY12 and the pan-macrophage marker F4/80. Imaging was performed using a Leica TCS SP8 confocal microscope. Given the difficulty in distinguishing monocyte-derived macrophages (MDMs) individually within the central lesion core at 28 dpi, the density of intraspinal P2RY12-F4/80+ MDMs across whole spinal cord sections was quantified as a percentage of the section area. For region of interest (ROI) analysis at the epicenter, 0.1 mm² boxes were positioned centrally on the section and over the ventromedial white matter. The P2RY12-F4/80+ staining area within each ROI was selected using the threshold tool in ImageJ and reported as a percentage of the ROI area. To evaluate the size of phagocytically active MDMs in the remaining ventral white matter, sections were immunostained with P2RY12, CD68, and myelin basic protein (MBP). Images were acquired using a Leica TCS SP8 confocal microscope. The spared ventral white matter was carefully outlined, and the surface area of individual P2RY12-CD68+ cells in this region was quantified using Imaris (totaling 177–395 cells per animal). The mean cell area in this region was calculated for each animal. In the case of crush SCI, the area occupied by F4/80+ cells in horizontal sections at the lesion epicenter was manually delineated in ImageJ and expressed as a percentage of F4/80+ tissue within a 2 mm-length area centered on the crush site.

**GFAP Intensity Analysis**

To measure the intensity of GFAP labeling, horizontal sections from mice with crush SCI were stained for GFAP. Images were captured using a Leica TCS SP8 confocal microscope and then converted to an 8-bit format. With the rostral side positioned to the left, a rectangular box centered on the crush site was drawn on the image. This box extended 1.5 mm along the rostral-caudal axis and reached up to the pia mater, ensuring that no background pixels were included. The grayscale profile along the horizontal axis was generated in ImageJ, and the data was subsequently exported to Microsoft Excel.

**Neurons**

To approximate the number of neurons in horizontal sections of compressed spinal cords, the sections were immunostained for neuronal nuclei (NeuN). Four regions of interest (ROIs), each 500 μm wide (designated as ROI #1–4), were placed on the section. ROI #1 was positioned starting at the lesion edge, which is defined by dense astrogliosis. ROI #2–4 began where the previous ROI ended, collectively spanning a total distance of 2 mm from the lesion edge. NeuN-positive cell bodies within each ROI were manually counted.

**Lipid Phagocytosis**

To quantify phagocytosed lipid debris, the Oil Red O staining method was employed. Sections were first rinsed in dH2O and 70% ethanol for 2 minutes each before being immersed in a saturated Oil Red O solution (catalog # O0625-25G; Sigma-Aldrich) dissolved in 70% ethanol at 60°C for 30 minutes. Following this, the tissue was differentiated in ethanol, washed with distilled water, and mounted with Immumount (Thermo Fisher Scientific). The area and number of phagocytosed lipid clusters were manually quantified using ImageJ.

**Axon Dieback**

Tiled montages of horizontal sections stained for BDA, GFAP, and F4/80 (with a depth of approximately 280 μm) were acquired using a Leica TCS SP8 confocal microscope. The retraction of BDA-positive dorsal CST axons from the edge of the T9 crush lesion (identified by GFAP labeling) was measured in one horizontal section per mouse using ImageJ. Additionally, higher magnification images of BDA-positive axons were captured.

**Inflammasome Activation and Inhibition**

Primary microglia were activated with LPS (100 ng/mL) for 3 hours in DMEM containing 10% FBS, followed by stimulation with 6-OAU (30 μM or as specified) in Opti-MEM medium (Life Technologies) for 30 minutes. Alternatively, the cells were treated with aluminum hydroxide (Alum, 150 μg/mL) or monosodium urate crystals (MSU, 500 μg/mL) along with 6-OAU (30 μM) for 4 hours. In experiments involving a GPR84 antagonist, CLH536, primary microglia were cultured under LPS stimulation, with CLH536 added 30 minutes before the introduction of 6-OAU (30 μM). After treatment, supernatants, cell lysates, and pellets were collected for subsequent ELISA and Western blot analyses.

**Adeno-associated Virus (AAV) Vector Production**

The AAV vectors used in this study (AAV-FKBP5, AAV-FXYD5, and AAV-lgals1) were produced and packaged by the Shanghai Genetic Chemical Corporation (PRC). The FKBP5, FXYD5, and lgals1 genes were cloned into the pAV-CMV-EGFP plasmid to generate pAV-CMV-FKBP5-EGFP, pAV-CMV-FXYD5-EGFP, and pAV-CMV-lgals1-EGFP, respectively. AAV9-CMV-FKBP5-EGFP, AAV9-CMV-FXYD5-EGFP, and AAV9-CMV-lgals1-EGFP were obtained by transfecting the respective plasmids, a helper adenovirus (AD) plasmid, and an AAV Rep/Cap-expressing plasmid into AAV-293 cells. AAV9-CMV-EGFP (empty vector) was produced similarly as a control. Viral particles were purified using the iodixanol gradient method. The titers of AAV9-CMV-FKBP5-EGFP, AAV9-CMV-FXYD5-EGFP, AAV9-CMV-lgals1-EGFP, and AAV9-CMV-EGFP were measured by quantitative real-time polymerase chain reaction (qPCR) to be 2.43 × 10^12, 2.35 × 10^12, 2.40 × 10^12, and 2.2 × 10^12 genomic copies per milliliter, respectively.

**Behavioral Evaluation**

Locomotor function was evaluated using the Basso Mouse Scale (BMS) on days 0, 1, 3, 7, 14, 21, and 28 post-SCI to assess functional recovery. BMS scores range from 0 to 9, where 0 indicates complete paralysis and 9 represents normal motor ability. Footprint analysis was conducted 28 days after the procedure. Different colored dyes were applied to the hindlimbs (marked in red) and forelimbs (marked in blue). Two researchers, unaware of the group allocations, independently assessed the results. Specifically, front paws were painted with blue dye and hind paws with red dye before allowing the mice to walk in a straight line on paper. Once the dye marks dried, a ruler was employed to measure relevant data, followed by statistical analysis of stride length and instances of toe dragging.

**Immunofluorescence (IF) Staining**

Samples were collected for immunofluorescence (IF) staining on days 1, 3, 7, 14, and 28 post-SCI. IF staining was performed on spinal cord sections rostral to the injury site (1 mm in length, located 4 mm from the injury center) as outlined previously. The protocol involved deparaffinizing, rehydrating, and washing the samples, followed by a 20-minute incubation in 10.2 mM sodium citrate buffer at 95°C. Slices were then permeabilized with 0.1% (v/v) PBS-Triton X-100 for 10 minutes. Subsequently, the slices were blocked with 10% (v/v) bovine serum albumin (BSA) in PBS for one hour before being incubated overnight at 4°C with primary antibodies against various markers including FKBP5(1:1000), GPR84(1:1000), LDHA(1:200), Fxyd5(1:200), Lgals1(1:1000), iNOS(1:500), TNF-α(1:500), Arg-1(1:1000), CD206(1:200), histone-H4(1:1000), IL-10(1:1000), RIPK3(1:300), C-Caspase-3(1:1000), C-Caspase-8(1:1000), MLKL(1:1000), Caspase-1(1:1000), GSDMD(1:1000) and IL-1β(1:1000). Following this, the samples underwent three 10-minute washes at room temperature, were incubated with secondary antibody for 1 hour at 37°C, and finally counterstained with DAPI solution.

**ChIP-qPCR**

The ChIP assay was conducted using the ChIP-IT® Express Enzymatic Magnetic Chromatin Immunoprecipitation Kit & Enzymatic Shearing Kit (Active Motif), following a previously described method. Briefly, isolated Pre and 14 dpi tissues or cultured primary microglia were crosslinked with 1% formaldehyde for 30 minutes at 37°C, followed by quenching with glycine for 10 minutes. The tissues or cells underwent three PBS washes and were centrifuged at 2000 rpm for 10 minutes, then lysed, sonicated, and immunoprecipitated according to the manufacturer’s instructions. The diluted chromatin was subjected to immunoprecipitation using H4K12la or PD-1 antibodies, while normal rabbit IgG served as a nonspecific control. The mixture was rotated continuously during overnight incubation at 4°C. Subsequently, 50 μl of protein A/G magnetic beads were added and incubated at 4°C for 2 hours. After washing the beads with wash buffer, the crosslinks were reversed by incubating them in 1% SDS and 0.1 M NaHCO3 at 50°C for 12 hours, as specified in the kit. DNA fragments were purified using the QIAquick PCR Purification Kit (QIAGEN). The ChIP-derived DNA was detected by qPCR. For normalization, a non‑regulatory intronic region of the Gapdh gene was used as a negative control locus. The primer sequences for the Fxyd5 promoter region were:

forward 5′‑ CATCTGCGTCCCTCAGATCC ‑3′ and

reverse 5′‑ GACCCATGGTTCCTGAGTGG ‑3′.

The negative control primers for the Gapdh intronic region were:

forward 5′‑ GGTTGCTGTGTCACTACCGA ‑3′ and

reverse 5′‑ GAGGGCTGCAGTCCGTATTT ‑3′. ChIP‑qPCR data were normalized using the "Fold Enrichment" method: First, the relative amount of the target region compared to the negative control region for each sample (ChIP and Input) was calculated using the 2^(‑ΔCt) method, where ΔCt = Ct[target] − Ct[negative control]. Then, Fold Enrichment = 2^(‑ΔCt[ChIP]) / 2^(‑ΔCt[Input]). This directly reflects the enrichment of H4K12la at the specific genomic locus relative to background and total input DNA.

**Detection of Lactate Levels**

Lactate levels in spinal cord tissues were measured following a previously described method. Briefly, spinal cord tissues were sonicated and homogenized in lysis buffer at 500 W (4 seconds on and 8 seconds off) for a total duration of 4 minutes. This was followed by centrifugation at 15,000 rpm for 15 minutes to obtain the supernatant. The lactate concentration was then determined using the ab65330 Lactate Assay Kit (Colorimetric/Fluorometric), according to the manufacturer’s instructions, with results expressed in mM.

**Cell Proliferation**

Sections underwent immunostaining for GFAP, NG2, and BrdU, followed by imaging with a Leica TCS SP8 confocal microscope. The counts of cells double-labeled with GFAP+BrdU+ and NG2+BrdU+ were performed at 0.6 mm anterior and posterior to the injury center, as well as at the epicenter itself, utilizing Imaris v8.0 (Bitplane Scientific Software). In the case of crush spinal cord injuries, this analysis was confined to the section at the injury epicenter. To achieve this, co-localization channels for GFAP/BrdU was established based on the absolute intensity of staining. Overlapping objects were separated, and the channel was transformed into a surface. Thresholds were determined according to cell area and the encapsulation of a BrdU-positive nucleus by either GFAP.

**Supplementary Figure 1. Quality control of single-cell sequencing data in spinal cord injury**

(A-B): Single-cell data before (A) and after (B) quality control, illustrating the impact of QC steps on data clarity and reliability.

(C): Identification of highly variable genes in single cells, which is crucial for distinguishing different cell types based on gene expression patterns.

(D): Principal component analysis (PCA) of single-cell samples for dimensionality reduction, which aids in the visualization of major variance directions and cellular population structures.

(E-F): Expression of marker genes across the cell types. Panel E shows the expression patterns of marker genes used to identify and distinguish between different cell types, whereas panel F displays the distribution of the expressions of these marker genes across different samples.

(G-H): Stacked bar charts showing the number of cells in different samples. Figure G illustrates the distribution of various cell types within each sample group, whereas Figure H compares the cellular compositions between different samples, providing insights into the changes between the experimental and control groups.

**Supplementary Figure 2. Immune cell composition at the spinal cord injury site**

This figure presents a UMAP heatmap based on the marker genes, revealing the characteristics of the immune cell composition at the site of spinal cord injury.

**Supplementary Figure 3. Microglia are essential for bridge formation and rapid healing post-spinal cord injury**

(A) Images of the spinal lesion area after injury, stained with specified antibodies or DAPI (blue) (scale bar = 20 μm).

(B) Representative images from Cx3cr1-GFP mice showing the spinal lesion site at various time points post-injury, stained with antibodies against P2Y12, SPP1, and DAPI (blue) (scale bar = 20 μm).

(C) Spinal sections from different groups of mice at 3 dpi, stained with indicated antibodies or DAPI (blue) (scale bar = 20 μm).

(D) Spinal sections from different groups of injured mice at 7 dpi, stained with antibodies against 5-HT, GFAP, and DAPI (scale bar = 20 μm).

(E) Spinal sections from mice at 3, 7, or 14 dpi, stained with antibodies against CD68 and P2Y12. High-magnification images revealing co-localization of P2Y12-positive cells with CD68 at 3 dpi. By 7 and 14 dpi, the cells around the lesion exhibit highly ramified morphologies (scale bar = 20 μm).

**Supplementary Figure 4. Histological evaluation of bridge structures following spinal cord injury**

(A) High-resolution images of the spinal cord sections from the bridging area at 3 dpi, which were stained with antibodies against fibronectin, GFAP, P2Y12, type I collagen, and DAPI (blue) (scale bar = 200 μm).

(B) Representative images of spinal cord sections from Cx3cr1-GFP mice at 1 and 3 dpi, labeled with Caspase-3, illustrating cells surrounding the lesion sites (scale bar = 200 μm).

All experiments depicted in this figure were independently replicated three times, yielding consistent results.

**Supplementary Figure 5. PANoptosis-like cell death occurs in neurons post-spinal cord injury**

(A-B) SCI-induced inflammasome activation: Representative immunofluorescence images (A) and quantification (B) of ASC speck formation in Sham versus SCI (1dpi). N=6. Scale bars: 10 μm.

(C) Co-immunoprecipitation (Co-IP) assays in WT, FKBP5−/−, and ASC−/− neurons: Anti-ASC pulldowns followed by immunoblotting for PANoptosome-related components (RIPK3, CASP8, NLRP3), confirming FKBP5-dependent complex assembly.

(D-E) The CCK8 (D) and LDH (E) kits were used to detect the activity and death levels of cells treated with different inhibitors (VX-765, Z-VAD-FMK, Necrostatin-1).

n = 3, Data are presented as mean ± SEM; **p < 0.01; ***p < 0.001.

**Supplementary Figure 6. Microglia regulate PANoptosis-like post-spinal cord injury via FKBP5**

(A) Representative images of the spinal cord frozen sections from each group, stained with NeuN (green), TUNEL (red), and DAPI (blue) (scale bar = 100 μm). Quantitative analysis of the TUNEL-positive neurons in the ventral horn region (n = 5).

(B) Representative images of the spinal cord frozen sections from each group, stained with NeuN (green), PI (red), and DAPI (blue) (scale bar = 100 μm). Quantitative analysis of the PI-positive neurons in the ventral horn region (n = 5).

(C) Co-immunoprecipitation (CO-IP) assay for examining the interaction between FKBP5 and IL-1β.

(D) CO-IP assay for examining the interaction between GPR84 and IL-1β.

n = 6, Data are presented as mean ± SEM; **p < 0.01; ***p < 0.001.

**Supplementary Figure 7. Lactate enhances the overall lactylation levels, improving the microglial phenotypes**

(A) KEGG pathway analysis of differentially expressed genes.

(B) Western blot analysis shows the protein lactylation levels across different experimental groups.

(C) Quantitative assessment confirms that lactate treatment significantly increases the overall protein lactylation levels compared to the control groups.

(D) Measurement of lactate concentrations in different experimental conditions demonstrates the effectiveness of lactate administration in achieving higher intracellular lactate levels, which may contribute to improved recovery outcomes.

(E) QPCR analysis examines the expression levels of key pro-inflammatory (IL-1β, iNOS, and TNF-α) and anti-inflammatory (Arg-1, CD206, and IL-10) markers.

(F) Immunofluorescence staining highlights the changes in the expression patterns of anti-inflammatory marker CD206 and pro-inflammatory marker iNOS in response to lactate treatment, visually demonstrating the shift from a pro-inflammatory to an anti-inflammatory phenotype (scale bar = 20 μm)

(G) Quantification of immunofluorescence signals further substantiates the observed changes in CD206 and iNOS expressions.

(H) Protein level confirmation through Western blotting reinforces the mRNA data, showing consistent downregulation of pro-inflammatory markers and upregulation of anti-inflammatory markers in the lactate-treated groups.

(I) Semi-quantitative analysis of protein levels for IL-1β, iNOS, TNF-α, Arg-1, CD206, and IL-10.

n = 6. Data are presented as mean ± SEM; *p < 0.05; **p < 0.01; ***p < 0.001.

**Supplementary Figure 8. FKBP5 promotes phosphorylation at the LDHA-Y10 site**

(A-B) Oxygen consumption rates (OCRs) in the microglial cells following FKBP5 knockdown.

(C) Western blot analysis of LDHA and LDHB protein levels after knocking down FKBP5.

(D-E) The lactate production and LDH enzyme activity in FKBP5-overexpressing or FKBP5-knockdown cells were examined under the indicated conditions.

(F) Whole-cell lysates from 3 × 10^6^ microglial cells stably transfected with control shRNA or shFKBP5 shRNA were subjected to gel filtration analysis. Western blot was used to assess the LDHA protein levels across different fractions collected via gel filtration.

(G) FKBP5 regulates the LDHA-Y10 phosphorylation in the microglial cells.

(H) FKBP5 modulates the LDHA phosphorylation in an FGFR1-dependent manner.

(I) Purified recombinant LDHA and FKBP5 proteins were incubated with or without active recombinant FGFR1 in an in vitro kinase assay.

(J) Immunohistochemical scores indicate a positive correlation between LDHA-Y10 phosphorylation and FKBP5 expression.

Data are presented as mean ± SEM; ***p < 0.001.

**Supplemental Figure 9. Differentially expressed genes in various immune cells**

(A) A volcano plot of differentially expressed genes in various immune cells.

(B) KEGG analysis of differentially expressed genes in the microglial cells.

**Supplemental Figure 10. Functional Validation of Lgals1 in Primary Microglia**

(A) The level of Lgals1 mRNA was detected by QPCR.

(B) ELISA detection of anti-inflammatory markers (e.g., Arg1, Cd206, IL-10) and suppressed pro-inflammatory cytokines (e.g., IL-1β, TNF-α).

(C) A microglia–neuron co-culture system;

(D) IF detection of neuronal neurite growth, further confirming its functional role (scale bar = 5 μm).

(E) ELISA detection of anti-inflammatory gene expression, further confirming its functional role.

n = 6. All data are presented as mean ± SEM; **p < 0.01; ***p < 0.001.

**Supplemental Figure 11. Microglia-dependent signaling networks in injured spinal cords**

(A) Cell communication, where the cell points indicate the number of cells and thickness of the lines represents the strength of communication;

(B-C) Single-cell communication;

(D) Receptor-ligand interactions between the microglia and macrophage with other cell types.

**Supplemental Figure 12. Microglia-dependent signaling networks in an injured spinal cord**

(A) A diagram of cell communication. The left side showing cell communication in untreated (uninjured) conditions and the right side showing cell communication post-injury.

(B) A diagram of receptor–ligand communication. The left side illustrates receptor–ligand interactions in the untreated (uninjured) conditions and the right side shows these interactions post-injury.

**Supplemental Figure 13. Violin plots showing ligand–receptor gene expression involved cell communication in an injured spinal cord**

**Supplemental Figure 14. GPR37 expression is regulated by FKBP5 at the transcriptional level**

(A-B) Overexpression of FKBP5 was achieved, and the expression of GPR37 was detected by QPCR (C) and WB (B).

(C) ChIP-qPCR assays confirmed enhanced H4K12 lactylation at the GPR37 promoter upon FKBP5 overexpression.

(D-E) Loss of GPR37 significantly reversed the reparative phenotypes induced by FKBP5—including anti-inflammatory IL-10 secretion (D) and promotion of neurite outgrowth (E).

n = 3, Data are presented as mean ± SEM; **p < 0.01; ***p < 0.001.

**Supplemental Figure 15. Microglial depletion worsens motor recovery and increases tissue pathology after SCI**

(A) Describes the experimental group allocations and treatment schedules.

(B-D) BMS scores (B), BMS subscores (C), and Horizontal ladder test results

(D). Data showing the impact of microglial depletion on motor recovery following SCI.

(E-F) Eriochrome cyanine staining, neurofilament staining, and quantification. Illustrates myelin and axonal integrity with associated quantifications (scale bar = 100 μm).

(G) Representative lesion center showing the microglia (P2RY12+ cells) and MDMs (P2RY12-F4/80+ cells). Provides a visual representation of the microglia and macrophage/dendritic cell populations within the lesion (scale bar = 100 μm).

(H-I) Effect of microglial depletion on MDMs. Microglial depletion reduces the overall MDM numbers but it allows these cells to migrate from the central gray matter (GM) into the spared tissue regions.

(J-K) Phagocytic MDMs in the ventromedial white matter at the lesion center (CD68 + P2RY12- cells). Representative images highlighting the phagocytically active MDMs (scale bar = 20 μm).

(L-N) Astrocytic borders at the lesion center (L). Displays representative astrocyte boundaries at the injury epicenter (scale bar = 100 μm). GFAP labeling along the spinal axis (M) and at the lesion center (N).

(O) A representative image of the proliferating astrocytes (arrows) in the ventromedial white matter (scale bar = 100 μm).

(P) Quantification of astrocyte proliferation.

n = 6. All data are presented as mean ± SEM; **p < 0.01; ***p < 0.001.

**Abbreviations:** AUC, area under the curve.
